# Generating multi-temporal landslide inventories through a general deep transfer learning strategy using HR EO data

**DOI:** 10.1038/s41598-022-27352-y

**Published:** 2023-01-04

**Authors:** Kushanav Bhuyan, Hakan Tanyaş, Lorenzo Nava, Silvia Puliero, Sansar Raj Meena, Mario Floris, Cees van Westen, Filippo Catani

**Affiliations:** 1 Machine Intelligence and Slope Stability Laboratory, Department of Geosciences, University of Padova, 35131 Padua, Italy; 2 Centre for Disaster Resilience, Department of Applied Earth Sciences, Faculty of Geo-Information Science and Earth Observation (ITC), University of Twente, 7514 AE Enschede, The Netherlands

**Keywords:** Natural hazards, Engineering

## Abstract

Mapping of landslides over space has seen an increasing attention and good results in the last decade. While current methods are chiefly applied to generate event-inventories, whereas multi-temporal (MT) inventories are rare, even using manual landslide mapping. Here, we present an innovative deep learning strategy which employs transfer learning that allows for the Attention Deep Supervision Multi-Scale U-Net model to be adapted for landslide detection tasks in new areas. The method also provides the flexibility of re-training a pretrained model to detect both rainfall- and earthquake-triggered landslides on new target areas. For the mapping, we used archived Planet Lab remote sensing images spanning a period between 2009 till 2021 with spatial resolution of 3–5 m to systematically generate MT landslide inventories. When we examined all cases, our approach provided an average F1 score of 0.8 indicating that we successfully identified the spatiotemporal occurrences of landslides. To examine the size distribution of mapped landslides we compared the frequency-area distributions of predicted co-seismic landslides with manually mapped products from the literature. Results showed a good match between calculated power-law exponents where the difference ranges between 0.04 and 0.21. Overall, this study showed that the proposed algorithm could be applied to large areas to generate polygon-based MT landslide inventories.

## Introduction

### Background

In mountainous regions, natural hazards such as landslides, avalanches, floods, and debris flows can cause significant property damage and human casualties^1^. Landslides are triggered by earthquakes^2–4^, extreme meteorological events such as intense precipitation^5^ and windstorms^6–9^, and anthropogenic activities^10^. Landslides alone have contributed to roughly $4.5 billion of economic loss between 1990 and 2017 according to the EM-DAT^11^, and approximately 58% of the fatalities and 69% of economic losses transpired in East and Southeast Asia, respectively.

In an attempt to understand the spatial and size distribution of landslides, polygon-based landslide inventories are very important as they form the basis for estimating the susceptibility^12^, hazard^13^, and risk^14^ as well as used in analyses for better understanding landscape evolution processes^15,16^ and for developing early warning systems^17^. Depending on the type of the inventory, we can record critical information about the spatial locations, the time of occurrence, area, and volume of landslides using inventories. For instance, many historical inventories lack information on landslide occurrence dates and corresponding triggering factors and yet, they still provide valuable information to generate landslide susceptibility map^18^. Landslide-event inventories, even if they are point-based products, carry information regarding the occurrence date of landslides and triggering factor^19,20^, which are essential for some applications such as developing a landslide early warning system or near-real-time predictive tools^21,22^. Polygon-based event inventories, on the other hand, could be used for rapid landslide hazard assessment^23^.

These inventories are generated using approaches such as (1) field surveys; (2) geomorphological mapping^24^; (3) visual image interpretation via sensors like Uncrewed Aerial Vehicles^25^, Light Detection and Ranging^26^, airborne instruments^27^, and satellite-borne antennas^28,29^; and (4) image processing tools for image classification and segmentation^30^. Despite all the methods developed and tested, there is still a lack in efficient and reliable methods to rapidly collect information on landslide occurrences. Specifically, accurate mapping of areal extents of landslides over large areas is still not a trivial task due to the inherent difficulties in manual mapping by expert geomorphologists as well as in semi-automated supervised mapping over the acquired image datasets^31^. As a result, most of the available landslide inventories are either historical (i.e., past and recent occurrences are all mapped together without information on activation time) or event-based products (i.e., only newly activated landslides are mapped as related to a triggering event, without reference to previous occurrences and possible reactivations of the same). This, in turn, hampers our capability of using slope instability as a measure of the slope response to seismic shaking or storms, and as a way, for instance, to explore sediment transfer from the headwaters down to the channel network and the depositional areas. What is needed for overcoming this limitation is an operational capability for an actual multi-temporal (MT) landslide mapping approach. A uniform and open accessible database information with polygon-based recorded landslides with information on the landslide type, component, and occurrence date for different parts of the world is required, particularly in the most recurringly susceptible areas.

MT landslide inventories can be defined as recurrent documentation of landslides, which could be independent of unique triggering events^32^. Multi-temporal mapping of landslides allows the generation of MT landslide inventories that updates historical landslide maps at multiple dates, which can be useful for supporting mitigation and adaption strategies such as monitoring susceptible areas^33,34^, developing dynamic landslide hazard assessment tools^35^ and/or exploring landscape evolution processes in response to climatic^36^, or seismic^3^ variables. MT inventories enable the identification of spatiotemporal patterns in landslide occurrences, which is a crucial pre-requisite for dynamic landslide hazard assessment, landslide mobility and evolution studies, and understanding the “legacy effects” of the triggering event^37^. Readily available and frequently updated MT inventories could also be used to support quantitative studies regarding the slope evolution after larger earthquakes or storms and in establishing baseline sediment mobilization volumes along a time line instead of at specific fixed times for landscape evolution modelling^38–41^. Recent studies have made important contributions in the field of post-seismic landslide evolution processes with the support of MT inventories in places including Wenchuan (Wenchuan earthquake), Indonesia (Reuleut earthquake), Papua New Guinea (Porgera earthquake), Collazzone region in Italy, and the Sulawesi: Kasiguncu and Palu earthquakes^42–45^. However, despite the useful information captured from MT inventories, they are quite rare because manually compiling MT inventories is a time-demanding process requiring high temporal resolution images. For example, there are only 12 cases where evolution of co-seismic landslides is monitored in post-seismic periods and more observations are mandatory to better understand landslide evolution processes^37,42^.

### Related work on automated multi-temporal landslide mapping

While there are works that touched on the topic of MT landslide mapping, the scale of mapping needs to be expanded and improved^32,46,47^. In the literature, object-based image analysis and Normalized Difference Vegetation Index (NDVI) thresholding was used to generate the MT inventories. The mentioned literature^32,46,47^ also refrained to only singular locations and did not test the capabilities on other sites or locations. Other studies also made great strides at mapping landslide masses temporally in Kyrgyzstan where their studies have shown the possibility of deriving long-term temporal landslide data^48,49^. An automatic method to generate MT landslide maps was proposed^32^, however, the approach relied on knowledge-based rulesets of landslide surface cover changes to map landslides. This knowledge-based system inhibits a “true” automated generation of MT inventories while also taking a much longer time in processing to detect landslides as they use bi-temporal satellite images coupled with NDVI threshold differences. Therefore, such a procedure can take quite some time to process and classify pixels into probable landslide candidates. Moreover, their approaches require a secondary step of converting pixel-based outputs into objects using the previously mentioned rulesets. This further hinders the transferability of such methods to other landscapes that could have varying topographic characteristics, thereby, simply relying on thresholding NDVI values might prove ineffective in mapping landslides spatiotemporally. Quite recently, advanced techniques emerging from the artificial intelligence (AI) domain have helped in developing new methods for rapid and accurate mapping of landslides. The ability to map landslides at different scales has become quite possible by using varying earth observation (EO) data in regions such as the Himalayas, the Japanese archipelagos, and South America comprising of countries like India, China, Nepal, Japan, and Brazil^30,50–55^. However, the shortage of high resolution (HR) satellite image data, issues of cloud obscuration, varying radiometric differences of landslides in image acquisitions, and the absence of adequate training samples are some of the reasons why it is difficult to train algorithms to detect landslides not just over space, but also over time^31^. The above-mentioned issues are prevalent even with conventional image processing techniques for mapping landslides, which implores us to envisage how challenging it is to map landslides in multiple temporal windows. Systematic temporal mapping of landslides has attracted scant attention in the AI domain. Landslides were mapped in Taiwan from 1998 to 2017 to monitor long-term landslides using a Random Forest classifier but were based on 30 m LANDSAT coarse resolution images^34^. A deep learning-based transfer learning approach was also devised with RapidEye high resolution images to map landslides both over space and time in Nepal for the 2015 Gorkha co-seismic landslides but the considered area of investigation was also relatively small^56^. A *practical*, *transferable*, *scalable*, and *automated* method to map landslides over different regions, between multiple temporal windows and also at varying scales has yet to be devised. The approach for global transferability will necessitate advanced methodological and technological developments in order to map landslides spatiotemporally for places with physical environments and landscapes that differ significantly^48^ and that is precisely what we aim to address in this study.

To address that point, we propose a novel automated deep transfer learning strategy that enables the mapping of landslides both over space and time. The model is also transferable and scalable, meaning that the trained models can be transferred to regions that vary topographically and efficiently generate MT landslides inventories.

### Study areas

**Figure 1:**
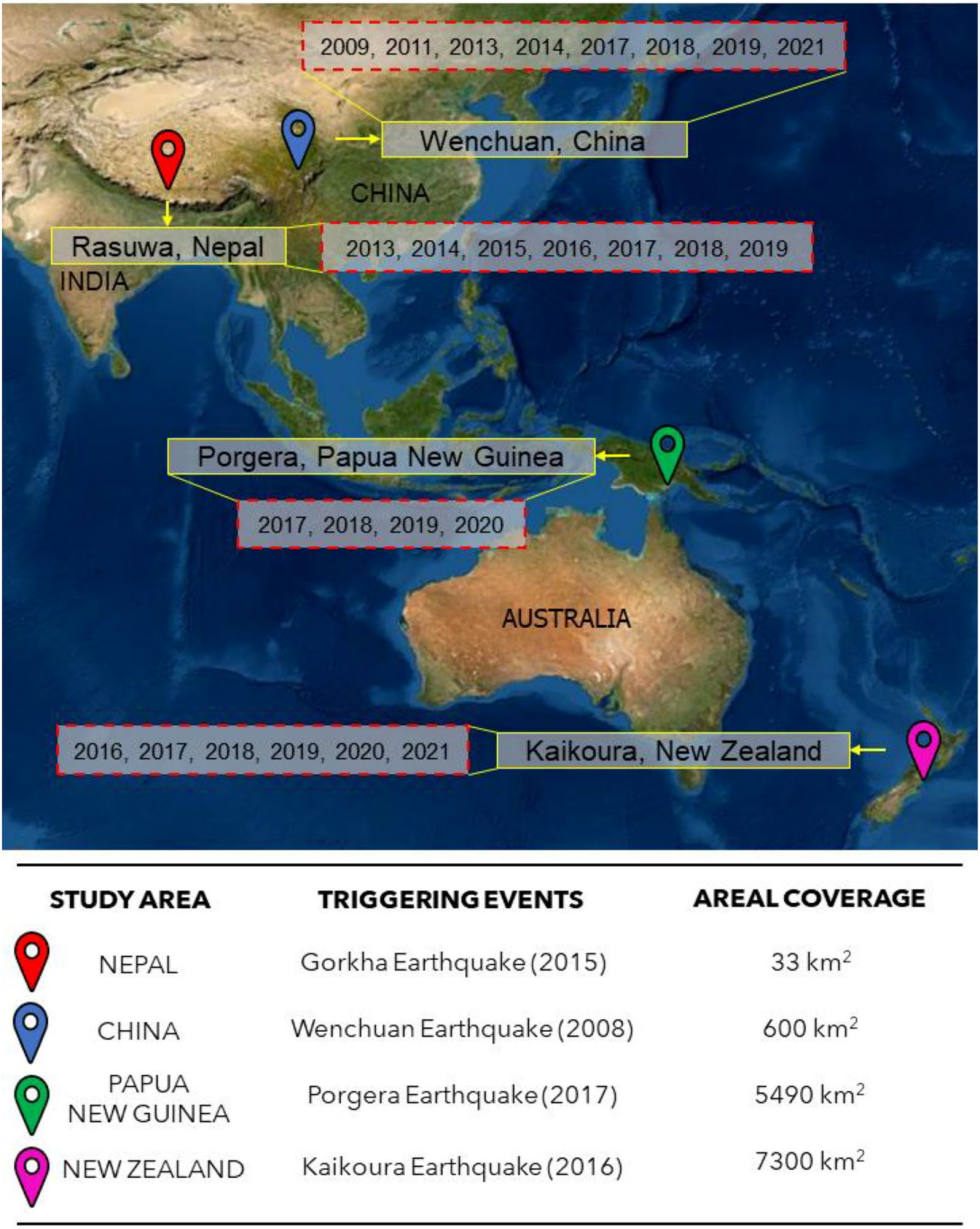
Overview of the study areas with the multi-temporal windows in the Asian-Pacific part of the world. Base map credits: www.arcgis.com/apps/mapviewer.

In order to calibrate and test the methodology, and to ensure diversity in the datasets, we take into consideration regions that went through some of the strongest episodes of earthquakes in the past years in several parts of the world, specifically the 7.9 Mw Wenchuan Earthquake (May 2008) in China, the 7.8 Mw Gorkha Earthquake (April 2015) in Nepal, and the 7.8 Mw Kaikōura Earthquake (November 2016) in New Zealand, and the 7.5 Mw Porgera Earthquake (February 2018) in Papua New Guinea (Fig. 1). These are all landslide prone areas hosting pre-, co- and post- seismic landslides. We examined subsets of these areas based on spatiotemporal availability of satellite images. Overall, we investigated a total of 13,423 km^2^ (including all four regions) for mapping landslides each year. Combining all the multi-temporal images, we mapped/detected landslides with a total areal extent of 70,791 km^2^ area. We examine these cases in ascending order with the smallest to the largest region as we want to initially develop our approach in a smaller domain and then apply/upgrade to larger areas.

### The 2015 Mw 7.8 Gorkha, Earthquake

**Figure 2:**
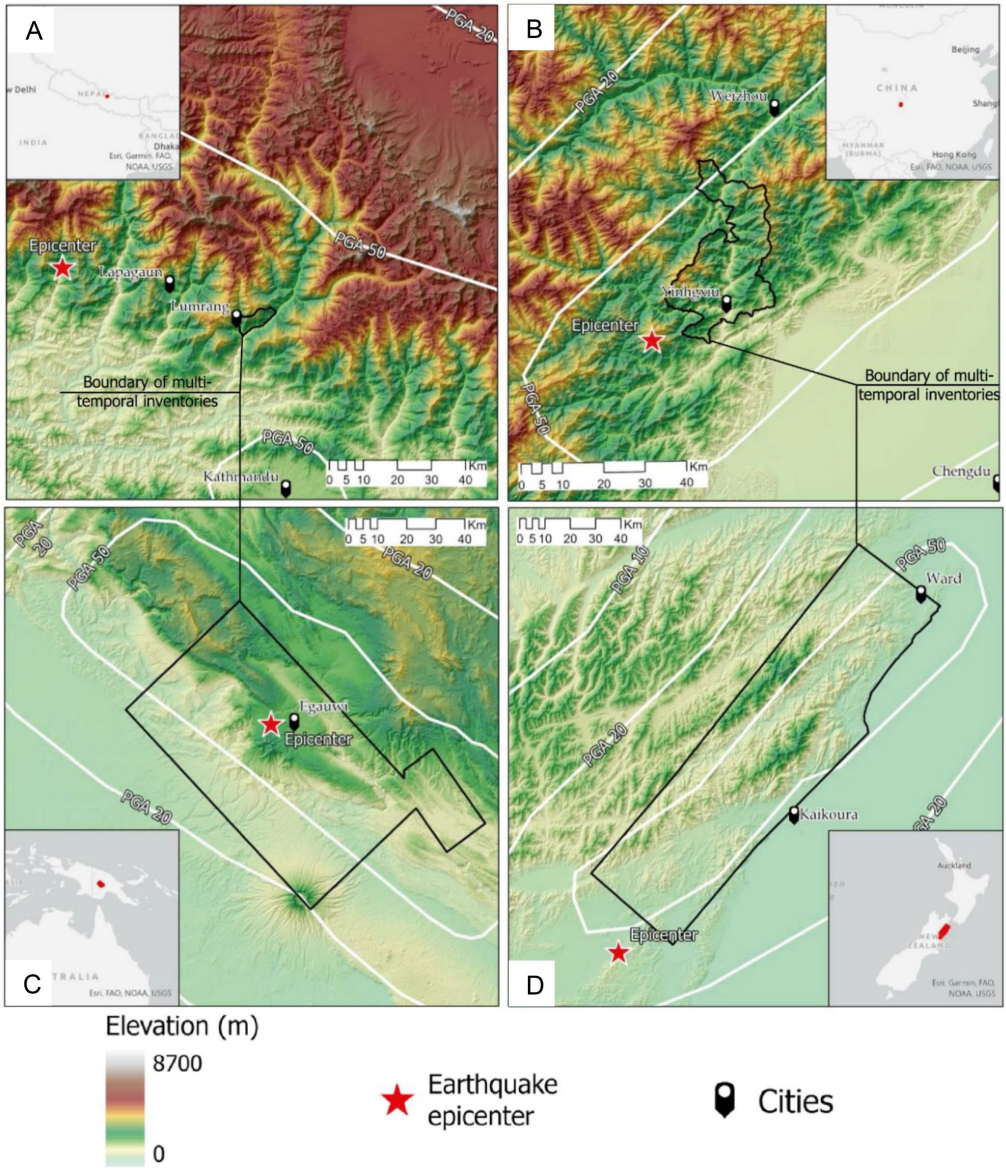
Study areas with the respective epicentres of the triggering events, the important cities within the vicinity of the investigation areas, and the PGA values (in percentage).

The study area (33 km^2^) is in the Rasuwa district of Nepal, along the Trishuli river with elevations ranging from 900 to 3250 m (Fig. 2a). The area has a temperate climate highly affected by monsoonal rains, with an annual average rainfall of 1800–2000 mm/year^57^. Geologically, the area is located in the Proterozoic Ranimata formation, which is dominated by phyllites, amphibolites, metasandstones and schists^58^ and the Ullrei formation, which consists of lenticular bodies of gneiss^59^, both of which are in the lower part of the Lesser Himalayan Sequence.

On April 25, 2015, the area was struck by the Mw 7.8 Gorkha earthquake, which triggered approximately 25,000 landslides^60^. The main shock was followed by an M_w_ 6.7 earthquake on the same day, an M_w_ 6.9 earthquake the day after and an M_w_ 7.3 earthquake on May 12, 2015. According to the United States Geological Survey (USGS), the main shock generated strong ground shaking reaching up to a maximum Peak Ground Acceleration (PGA) of 0.87 g^61^. Various studies examined the spatial distribution of co- and/or post-seismic landslides^35,62,63^ as well as their evolution over time via MT landslide inventories^36,56,64,65^. Here, we only focus on a subset of the area affected by the co-seismic landslide event (Fig. 2a), investigated in another study^56^ for MT inventory generation. However, the current work promotes updated products than the previous study as this research employs a much advanced and richer model.

### The 2008 Mw 7.9 Wenchuan Earthquake

The study area (600 km^2^) is located in the Wenchuan County, Sichuan Province of China, with altitudes ranging from 420 to over 4000 m (Fig. 2b). The area has subtropical climate influenced by monsoons, with a mean annual precipitation of more than 1250 mm/year^66^. Rocks from Sinian to Triassic age outcrop in the area. In particular, metamorphic rocks, such as schists and shales, sedimentary rocks, namely sandstones and limestones and volcanic rocks including granites and diorites are present^66^.

On May 12, 2008, the area of China was hit by the Mw 7.9 Wenchuan earthquake. The main shock, in which PGA values reached up to 1.14 g^67^ triggered approximately 200,000 landslides^68^. This became not only the world’s largest recorded landslide event but also one of the most studied case^44^. In addition to co-seismic hillslope failures, their post-seismic evolutions were also examined and documented in several studies^42,66,69^. This study focus on a specific subset of the earthquake affected area (Fig. 2b) in which MT landslide inventories are already available in the literature^66^.

### The 2018 Mw 7.5 Porgera Earthquake

The study area (5490 km^2^) is located in the central part of New Guinea island (western part of Papua New Guinea) (Fig. 2c). The elevation ranges from 140 and 3350 m. The study area has a monsoonal and tropical climate zone, with a mean annual precipitation of more than 2800 mm/year^70^. From a geological point of view, it is characterized by sedimentary rocks such as limestones, and sandstones and intermediate to mafic volcanic rocks from Oligocene to Pleistocene.

The M_w_ 7.5 Porgera earthquake hit the area on February 25, 2018. The main event was subsequently followed by four aftershocks with magnitudes (M_w_) greater than 6.0 in the next nine days. PGA values measured in the study area were up to 0.92 g^71^. The mainshock triggered more than 10,000 of landslides and the event was documented as one the of biggest landslide events of the last few decades^3^. Post-seismic evolution of landslides was examined via MT landslide inventories for only a small subset of the entire area affected by co-seismic landslides. In this study, we target a larger subset of the area to generate MT inventories (Fig. 2c) to cater to the availability of cloud-free images for landslide detection purposes.

### The 2016 Mw 7.9 Kaikōura Earthquake

The study area (7200 km^2^) is located in the Kaikōura region (South Island, Fig. 2d). The elevations range from 0 to 2850 m and have a temperate climate with mean annual precipitation between 500 and 1500 mm/year^72^. The geological units of the area include sedimentary and volcanic rocks from the Early Cretaceous to Quaternary period.

A strong earthquake of M_w_ 7.9 occurred on the island on November 14, 2016. Four aftershocks of M_w_ 6.0–6.5 struck the area in the following 13 h. PGA values reached in the area after the mainshock was up to 1.08 g according to^73^ and the earthquake triggered approximately 30,000 landslides^74^. However, the area still lacks MT landslide inventories possibly shedding light on post-seismic landslide evolution processes. Therefore, in this research, we generate MT inventories for the first time for a subset of the area affected by the Kaikōura earthquake (Fig. 2d).

## Results

### Generation of MT landslide inventories

**Figure 3:**
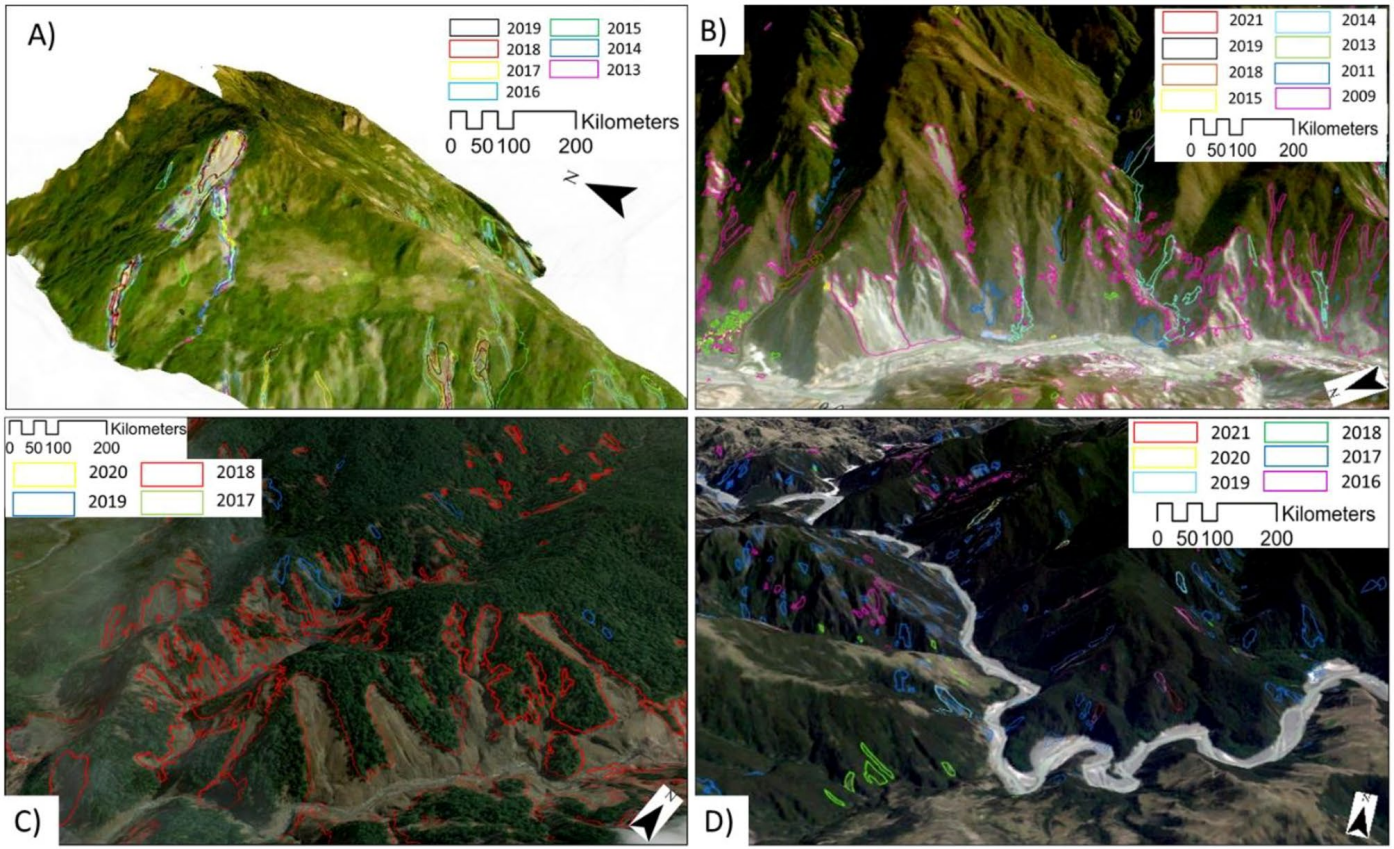
3D oblique view examples of temporal evolution of the landslides in (**A**) Nepal, (**B**) China, (**C**) Papua New Guinea, and (**D**) New Zealand.

Figure 3 shows three-dimensional examples of the post-seismic landslides in selected landscapes from (A) Rasuwa, Nepal, (B) Wenchuan, China, (C) Poregera, Papua New Guinea, and (D) Kaikōura, New Zealand cases. We can see that many new landslides occurred in the form of reactivations and remobilisation stemming around/from the older landslides. Ideally, classifying these reactivations and remobilisation based on the level of activity^75^ such as continued reactivations (the same material resumes or continues to fail), local activations (a previous slide alters the nearby hillslope, causing a subsequent slide), and remote activations (a previous slide generates changes elsewhere in the terrain that result in a later landslide) could aid in the later stages of dynamic landslide predictive modelling however, we do not distinguish them in this study as it is out of scope.

**Figure 4:**
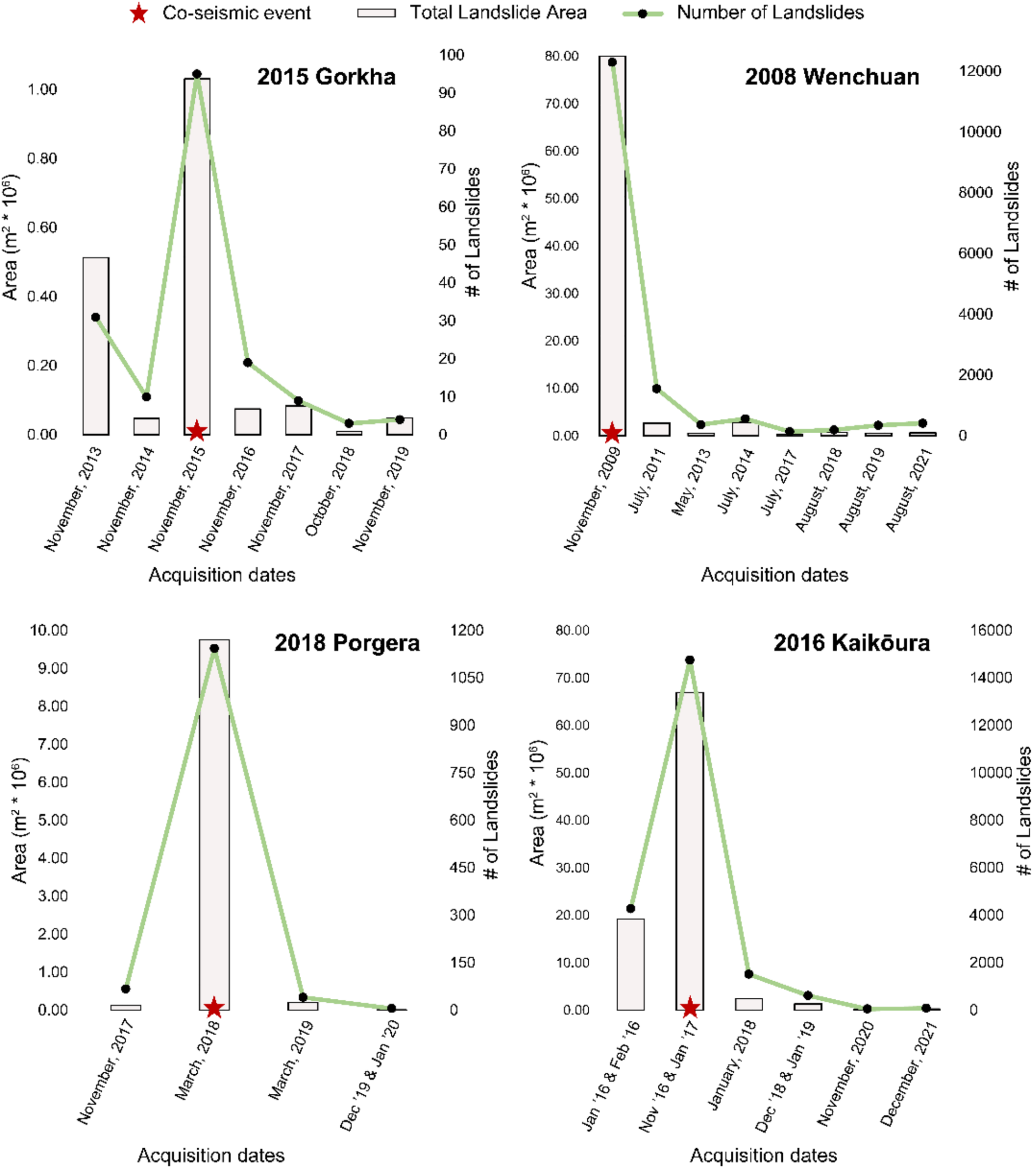
Trend of number of landslides and area of landslides through years for each study area detected by the model.

Most of the post-seismic landslides are smaller in size than the co-seismic landslides and the observations are comparable to the literature^42^ where new landslide occurrences are outside the co-seismic areas and a decay in the landslide activity is witnessed (Figs. S5, S6, S7, and S8). We see this decline in Fig. 4 where the number of landslides reduce significantly in the post-seismic years accompanied by a consistent decrease in the area of the landslides.

The temporal evolution of the mapped landslides for each study area can be seen in Figs. S5, S6, S7, and S8 in the Supplementary Materials. We observe the spatial distribution of the newly occurred, reactivated, and remobilised landslides after the main seismic event. Reminder that we do not separate the new (post-seismic) landslides based on the activity but simply comment/hint on the new failures in our generated inventories as it is out of scope for this research.

### Rasuwa, Nepal

The experiments start with the Gorkha case, which is the smallest location in terms of the mapping area and we move to the other regions of Wenchuan, Papua New Guinea, and Kaikōura to show the transferability and scalability of the transfer learning approach. The model is trained on the co-seismic inventory and then evaluated on a test set. In Table 1, the Precision, Recall, and F1-scores for each of the pre-, co-, and post-seismic inventories can be found. Overall, the F1-scores remains fairly well above 0.8 for the co-seismic and post-seismic inventories, however, for the pre-seismic inventories, the F1-scores are below 0.8. This can be explained because of existing satellite imagery artefacts like shadows, cloud-cover, haze, atmospheric disturbances, and image discoloration that confuses the model during the training phase and hence, affects the prediction towards the end. Details of the number of predicted/mapped landslides are also presented in Table 1.

**Table 1:** Accuracy of mapped landslides in time in Rasuwa, Nepal.

Year	Loss	Precision	Recall	F1-score	# of mapped landslides
2013	0.360	0.677	0.769	0.720	35
2014	0.381	0.595	0.770	0.671	21
2015*	0.297	0.767	0.841	0.801	98
2016	0.215	0.801	0.896	0.847	28
2017	0.218	0.790	0.887	0.835	18
2018	0.224	0.777	0.885	0.826	12
2019	0.226	0.771	0.887	0.825	24

*Co-seismic year.

**Figure 5:**
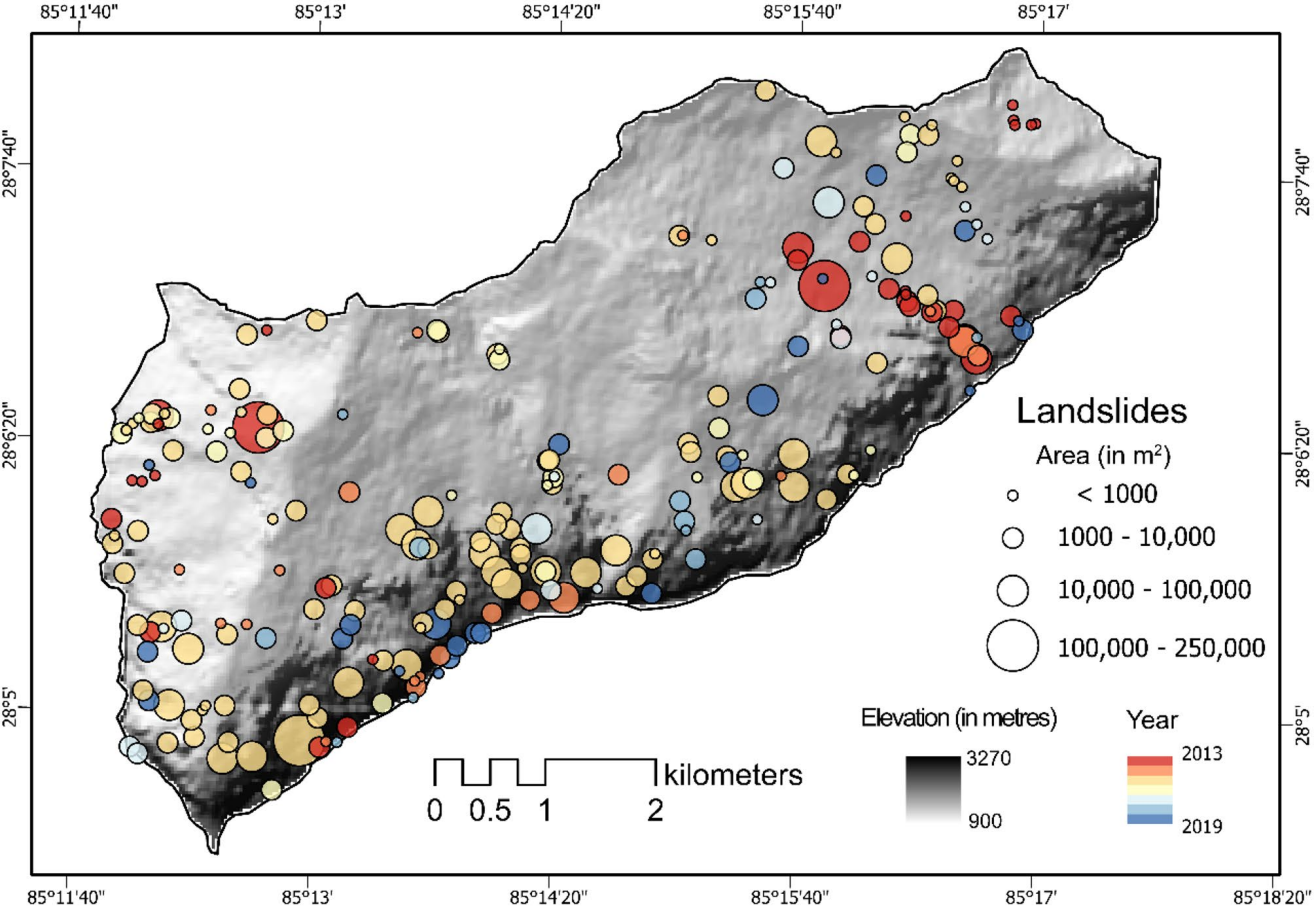
Multi-temporal landslide inventory of Rasuwa, Nepal from 2013 till 2019. The location, area (in m^2^), and the year of the inventories are depicted above (see Table S1 in the Supplementary Materials for the landslide statistics indicating differences between the GI and the PI for each year in the testing windows). Shaded relief base map credits: World Shaded Relief-ESRI.

In Gorkha, we delineate 35, 21, 98, 28, 18, 12, and 24 landslides temporally by the model for each of the years between 2013 until 2019, respectively. The average size of the co-seismic landslides is 11,465 m^2^ followed by the pre-seismic year of 2013 (8804 m^2^) while the rest remain fairly under 3500 m^2^ and the overall mean landslide size for all the years is 7865 m^2^. A reason for 2013 having such a huge mean landslide area is the presence of historical landslides in the region prior to the Gorkha earthquake event, while the latter years have considerably smaller mean area. We see a significant drop in the count as well as the average area of landslides after the event. Except for the co-seismic landslides triggered by the 2015 earthquake, precipitation is the main factor causing landslides in the latter years^64^. Most of the false positives are observed at the south-western part of the study area where the spectral signatures are similar to that of the landslides. Figure 5 shows the MT inventory for the Rasuwa district of Nepal where landslides occurred during the pre-, co- and post- seismic periods.

### Wenchuan, China

The region of the Wenchuan affected by the earthquake that we took into consideration is around 600 km^2^. The experiment of transferring the knowledge from Nepal to China was performed to improve the generalization of the Nepal trained model for locating landslides in China. As discussed previously, we use the trained model weights from the co-seismic year of Rasuwa, Nepal and then re-train on the co-seismic year of 2009 of Wenchuan, China. The updated model consisted of information of landslides from both Nepal and China. Although the Nepalese area is much smaller than the Chinese, the transfer learning process may still be considered valuable, owing to their similarities in geology and geomorphology. Table 2 shows the Precision, Recall, and F1-scores for the years between 2009 and 2021. The table also reports the number of mapped landslides by the model in the years between 2009 and 2021, with most of the landslide occurrences naturally observed in 2009. The mean average size of the co-seismic (2009) landslides are 6500 m^2^ followed by 5077 m^2^ of 2014, 3986 m^2^ in 2018, and the rest are below 2000 m^2^. The overall average landslide size over the years is 5563 m^2^.

**Table 2:** Accuracy of mapped landslides in time in Wenchuan, China.

Year	Loss	Precision	Recall	F1-score	# of mapped landslides
2009*	0.165	0.861	0.929	0.855	10,355
2011	0.207	0.828	0.867	0.846	1552
2013	0.107	0.986	0.959	0.934	370
2014	0.188	0.828	0.895	0.857	560
2017	0.208	0.779	0.899	0.834	140
2018	0.254	0.776	0.844	0.807	198
2019	0.380	0.864	0.764	0.810	349
2021	0.127	0.930	0.950	0.940	414

*Co-seismic event year.

**Figure 6:**
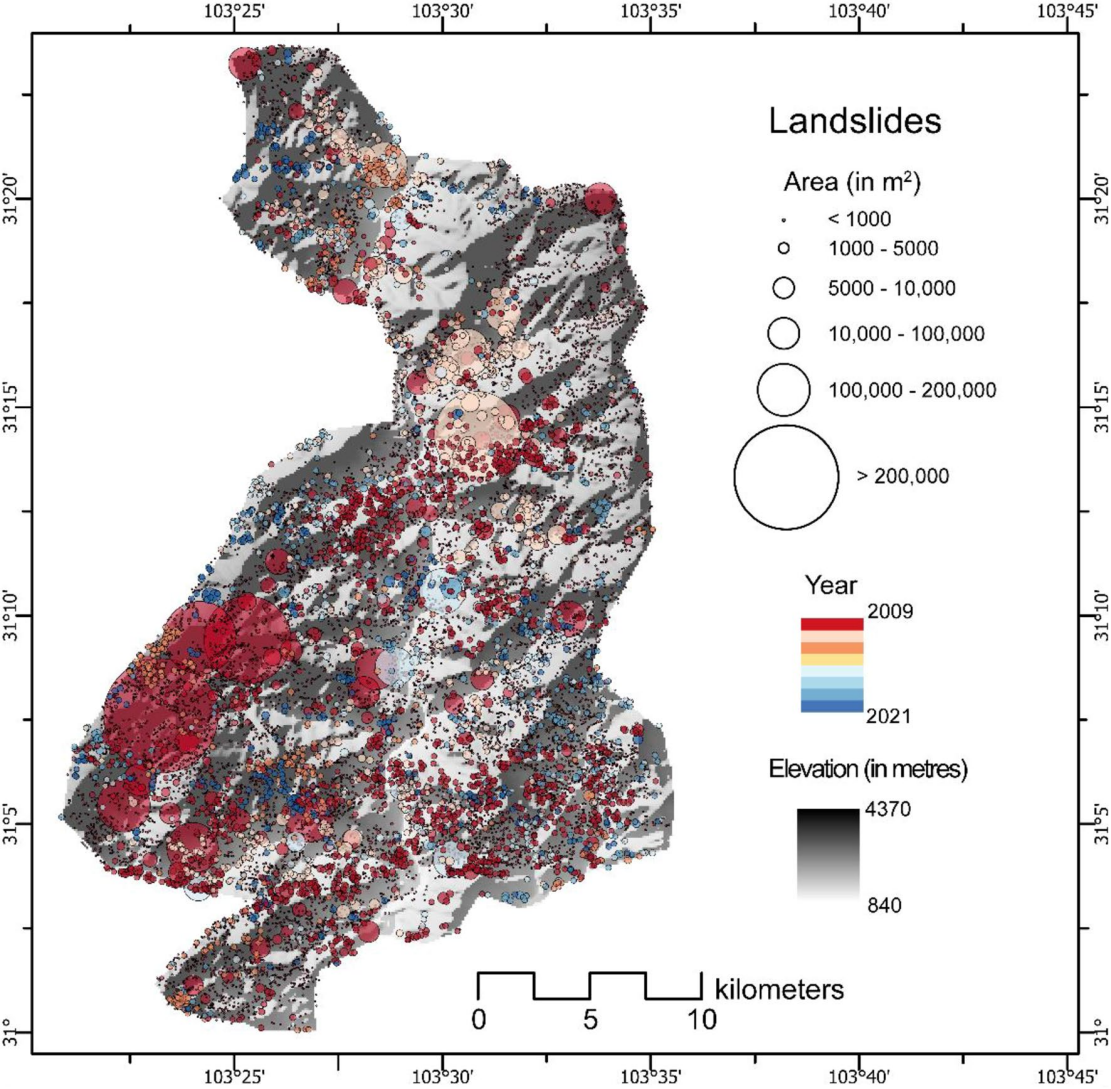
Multi-temporal landslide inventory of Wenchuan, China from 2009 till 2021. The location, area (in m^2^), and the year of the inventories are depicted above (see Table S2 in the Supplementary Materials for the landslide statistic indicating differences between the GI and the PI for each year in the testing windows). Shaded relief base map credits: World Shaded Relief-ESRI.

We notice that the overall F1-scores of the Wenchuan data in the testing windows depict very good scores, noticeably over 0.8, whereby in some cases even increasing above 0.9. This is due to the fact that in some of the years (2013 and 2021), the satellite images contain very low clouds and shadows, thereby accurately predicting the landslides when comparing to the reference ground truth. Figure 6 shows the temporal evolution of the landslides in the affected area from 2009 until 2021. The 2009 (few months after the 2008 event) predictions very well captured the landslides that occurred in the study area. The years for which the inventories were generated reflect HR satellite imagery availability.

### Porgera, Papua New Guinea

We now repeat the same approach that was applied to Wenchuan and bring in the weights of the model that were trained on China (with knowledge from Nepal) to re-train on the co-seismic year of Papua New Guinea. The updated model consists of a dataset including landslide information of Nepal, China, and Papua New Guinea, thereby, having a more comprehensive knowledge of landslides from three geographically distinct areas. Approximately, a total of 5490 km^2^ area was mapped for Papua New Guinea.

**Figure 7:**
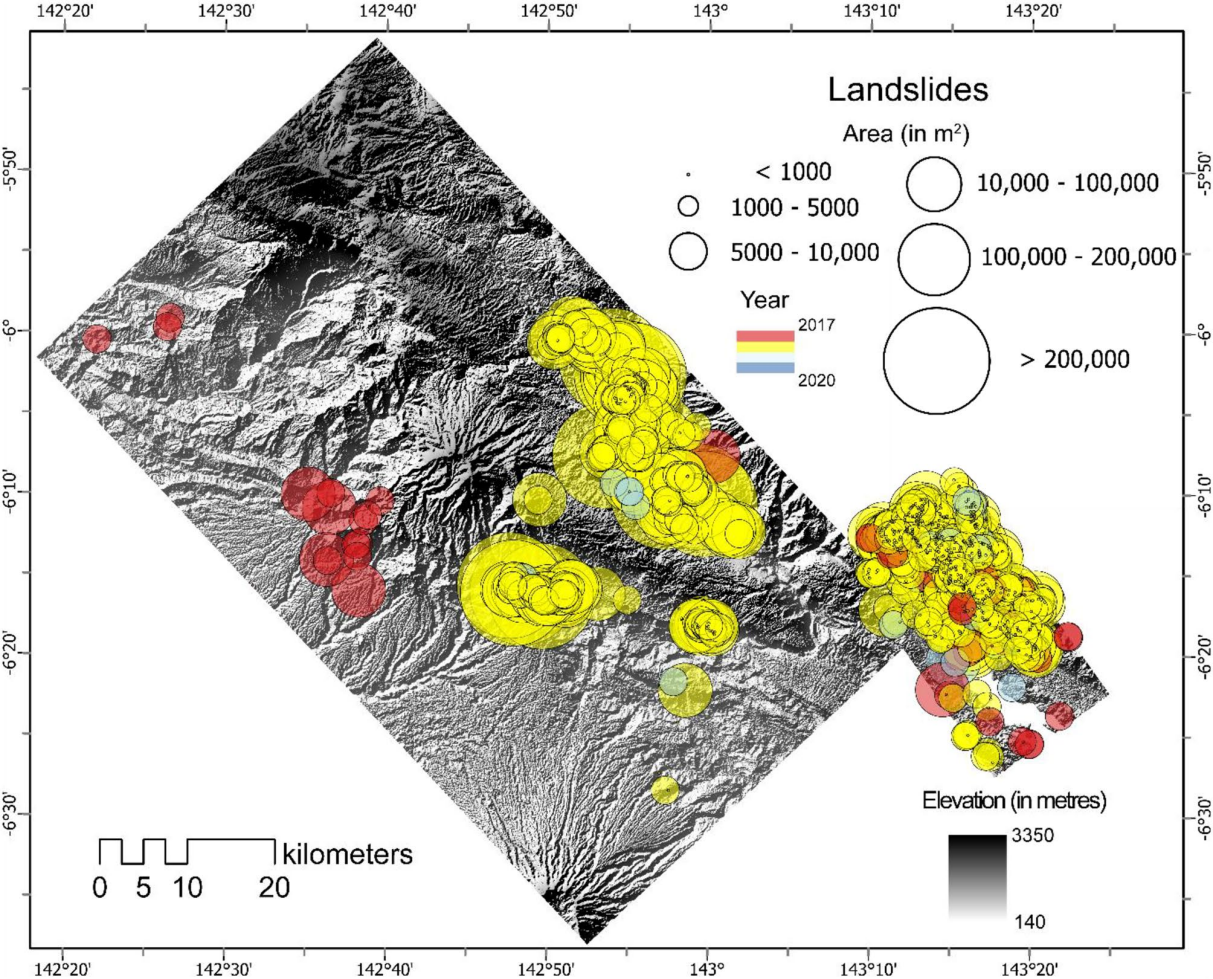
Multi-temporal landslide inventory of Porgera, Papua New Guinea from 2017 till 2020. The location, area (in m^2^), and the year of the inventories are depicted above (see Table S3 in the Supplementary Materials for the landslide statistic indicating differences between the GI and the PI for each year in the testing windows). Shaded relief base map credits: World Shaded Relief-ESRI.

Table 3 depicts the model performance on the test windows of Papua New Guinea via the metrics of Precision, Recall, and F1-scores. Overall, except for the year 2017, the F1-score is over 0.8 for the years 2018, 2019, and 2020. The reason for 2017 obtaining lower metrics, specifically lower Precision (0.68), is that due to the prevalence of more clouds, the model tended to predict these shadows as landslides, inadvertently increasing the FPs and thereby, reducing the Precision. The table also portrays the number of landslides that were detected in time. Although we see a much lower number of landslides in the post-seismic years (Fig. 7), it should be noted that the lack of available cloud-free images hinders the capability of the model to predict landslides under cloud obscuration. The average mean size of the co-seismic landslides area is 17,011 m^2^ while the pre-seismic year of 2017 has an average size of 15,000 m^2^ (which can be attributed to the presence of historical landslides). The two post-seismic years have a similar mean size of about 2050 m^2^ and the total average mean landslide size is 9028 m^2^.

**Table 3:** Accuracy of mapped landslides in time in Porgera, Papua New Guinea.

Year	Loss	Precision	Recall	F1-score	# of mapped landslides
2017	0.365	0.683	0.792	0.732	95
2018*	0.129	0.818	0.899	0.853	1557
2019	0.235	0.843	0.844	0.843	75
2020	0.123	0.896	0.805	0.846	5

*Co-seismic event year.

### Kaikōura, New Zealand

Finally, we take the trained weights from the previous three study areas and re-train in Kaikōura, New Zealand. Information about landslides from all the four study areas helps the model to inference on the entire study area more effectively. We see that on the derived metrics, specifically the F1-score that is usually above 0.8 except for the years 2016 and 2018 (see Table 4). Kaikōura has been the most challenging area to train the model on. This is because most of the terrain environment in the north and north-eastern part encompassed bare soil and rugged mountain topography. Most of the landslides also occurs in this type of terrain, therefore, leading to quite a bit of obvious FP predictions. Moreover, the model has been trained with proper knowledge of landslides within and around the peripheries of vegetation cover but since the geographical environment of New Zealand is much different than that of Nepal, China, and Papua New Guinea, the model performs relatively poor by over predicting and also under predicting many of the landslides.

**Table 4:** Accuracy of mapped landslides in time in Kaikōura, New Zealand.

Year	Loss	Precision	Recall	F1-score	# of mapped landslides
Early 2016	0.399	0.908	0.647	0.756	4266
Late 2016*	0.288	0.859	0.777	0.814	14,750
2018	0.358	0.826	0.685	0.746	1511
2019	0.179	0.848	0.899	0.873	613
2020	0.251	0.814	0.861	0.836	46
2021	0.156	0.834	0.930	0.877	82

*Co-seismic event year.

**Figure 8:**
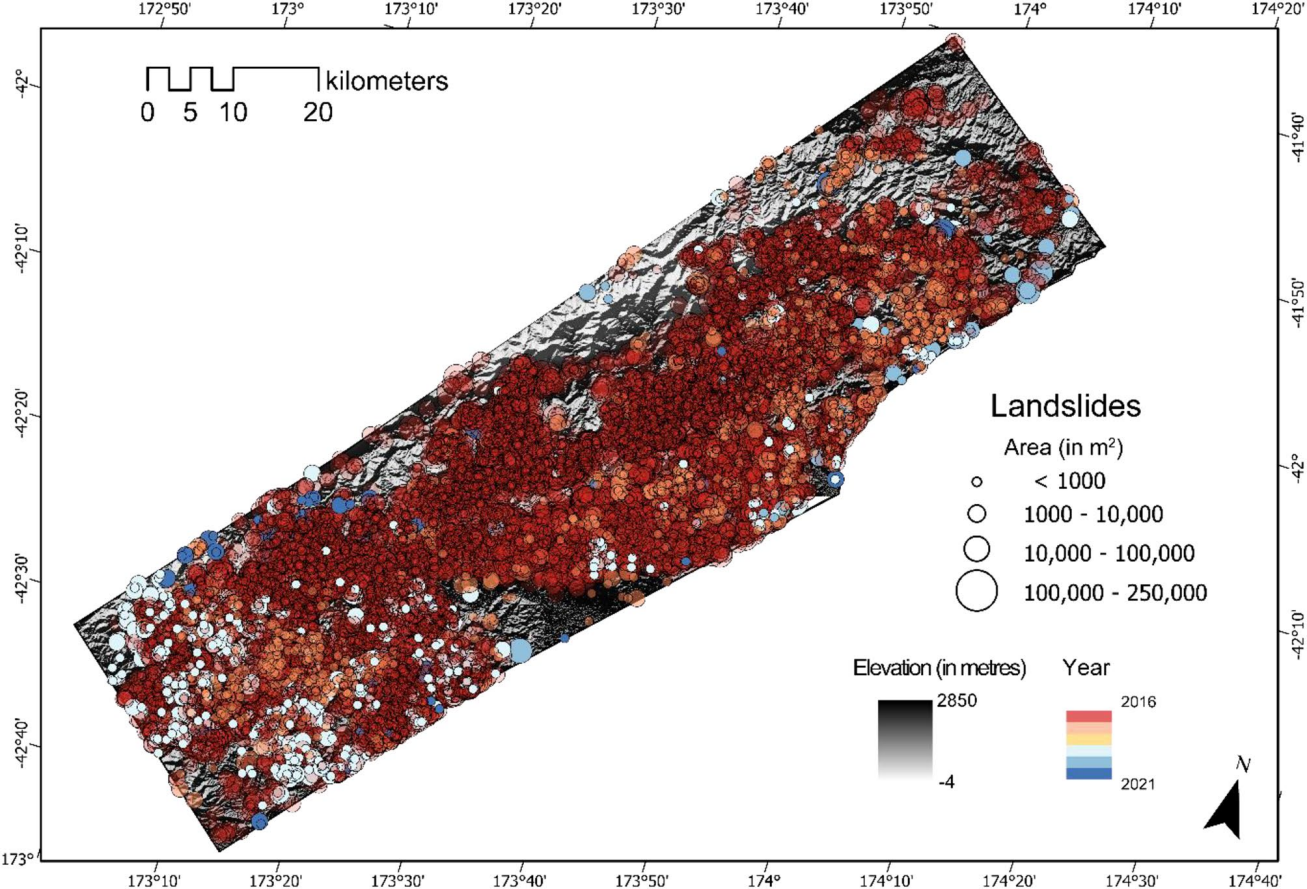
Multi-temporal landslide inventory of Kaikōura, New Zealand from 2016 till 2021. The location, area (in m^2^), and the year of the inventories are depicted above (see Table S4 in the Supplementary Materials for the landslide statistic indicating differences between the GI and the PI for each year in the testing windows). Shaded relief base map credits: World Shaded Relief-ESRI.

The number of modelled (or mapped) landslides in the co-seismic year was 14,750 and its number reduced dramatically in the post-seismic years as we also observed in other cases presented above. The mean area of the co-seismic landslides is 4535 m^2^ while the landslide area of the post-seismic years revolve around 2000 m^2^. The overall mean landslide area of the MT inventory is 3342 m^2^. Figure 8 shows the multi-temporal inventories generated by the model.

### Landslide area statistics

Landslide inventories not only include the spatial location and extent of the landslides but also other geometrical attributes like the area and perimeter. The area of the landslides is a fundamental metric for deriving information such as the volume of landslides which allows to estimate the intensity of the phenomena for zonation and hazard assessment^76^. To examine the size distributions of the generated MT inventories, we first plotted FAD curves.

**Figure 9:**
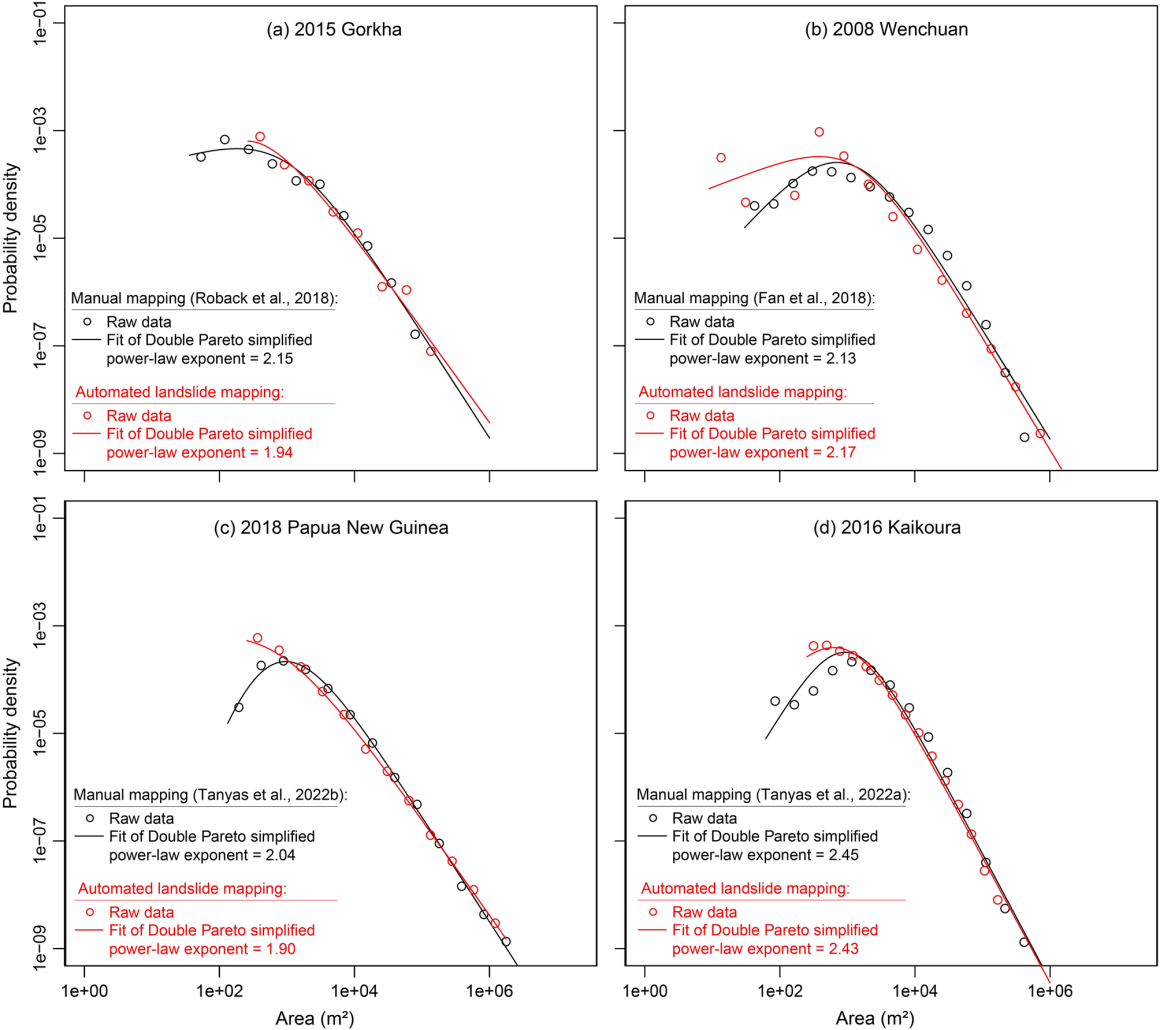
Frequency Area Distribution of the mapped inventories from the literature versus the prediction inventories for the four study areas. Note that the amount of data in 2015, Gorkha *might* be insufficient to delineate clear patterns and identify a roll-over point.

Figure 9 shows that the FAD curves for the automated mapping and the ground truth (from the literature) of the co-seismic years are very similar to each other (reminder that the ground truth inventory here is for the entire study area, not the testing subsets). The power-law exponents of corresponding inventories numerically show the similarity for each case (Gorkha: 1.94–2.15; Wenchuan: 2.17–2.13; PNG: 1.90–2.04 and NZ: 2.43–2.45, Fig. 9).

Overall, the fitted curve of both the automated and ground truth co-seismic inventories are pretty similar, which is a good sign in terms of looking at how close the predicted landslides are to the ground truth inventories. We do however see that post roll-over, the fitted curve of the automated inventory halts early for all except China. A reason for the early halt is that the model was not able to predict smaller landslides as compared to the manually mapped landslides of the ground truth inventories from the literature. The smallest area mapped by the model for China is similar to that of the reference ground truth. Although it could be said that this is because the model was able to detect the smaller landslides in China, it could also be that the population of the small area FPs mapped by the model was more in number (as we have a higher population of co-seismic mapped landslides, refer to Table S2 for the difference between co-seismic GI and PI). The minimum size of the landslides according to the FAD in Fig. 9 is as follows:

1. Papua New Guinea: *Automated Mapping* ~ 500 m^2^ and *Ground Truth* = 100 m^2^.
2. New Zealand: *Automated Mapping* ~ 100 m^2^ and *Ground Truth* = Less than 100 m^2^.
3. Nepal: *Automated Mapping* ~ 500 m^2^ and *Ground Truth* ~ 80 m^2^.
4. China: *Automated Mapping* ~ 20 m^2^ and *Ground Truth* ~ 80 m^2^.

We should also note that the manually generated inventories may have a larger uncertainty in the left part of the FAD, as mapping small-size landslides is always prone to interpretation error even when done by expert geomorphologists^24^.

For the Papua New Guinea region, there is a negligible difference in the power-law exponent value between the GI and the PI. This can be attributed to the fact that many of the landslide geometry were not mapped according to the manual ground truth inventory as seen in Fig. 11 (blue boxes), which is recognised to have experienced superficial sliding after the initial rotational failure. Thus, we witness spectral signatures pertaining to that of vegetation and therefore, the model does not classify these pixels as landslides.

**Figure 10:**
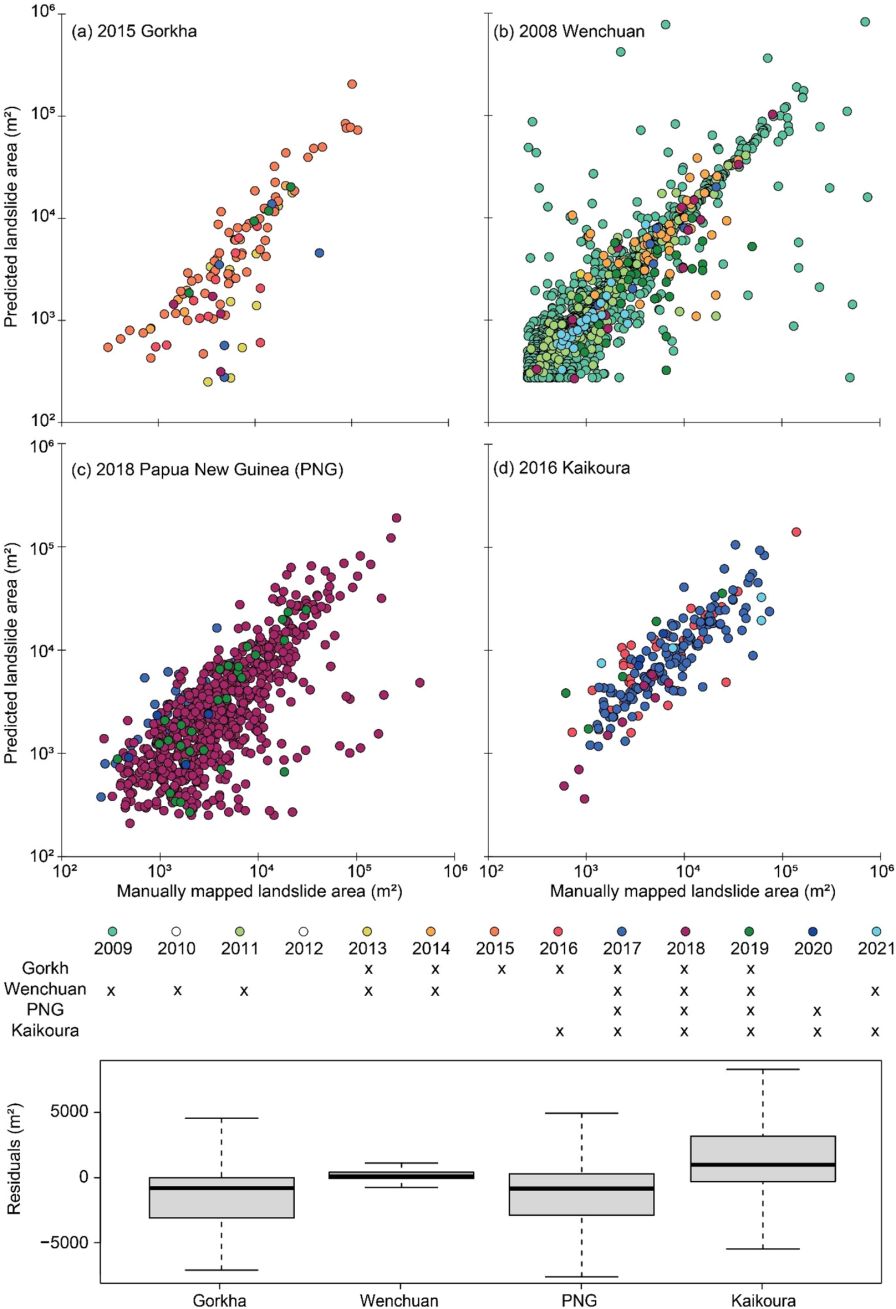
(Upper panel) Difference in area between the predicted landslides versus ground truth landslides. (Lower panel) Residuals measured in m^2^ between the ground truth landslides and predicted landslides.

To explore the size distribution landslides in terms of accuracy of delineated landslide polygons instead of the size statistics of all landslides, we ran further analyses. Figure 10 reports the scatter plot depicting the area of landslides in the MI in the x-axis and PI in the y-axis, supported by the residuals from the observations of the GI versus the PI. Results show that the residual (measures in m^2^) spread/distribution is very small and this implies very good prediction capability of the model. Specifically, the error of prediction in area has the error below ± 3000 m^2^ (i.e., error between the first and third quartile, see Fig. 10). This means that the error is mostly limited by 5–6 pixels in a DEM with a resolution of 1 arc-second. Moreover, the highest median of the residuals is around ± 1000 m^2^. In case of China, the residual (measures in m^2^) spread/distribution is very small and depicting very good prediction capability of the model. The same however, cannot be said for the other three regions. The error of prediction in area is reasonably good with the error under ± 5000 m^2^ (i.e., error between the first and third quartile, see Fig. 15). Moreover, the highest median of the residuals is around ± 1000 m^2^. The average mismatch between the areal extents of landslide polygons is around 700 m^2^, with the first and third quartiles of mismatching areas having less than 3000 m^2^.

We also checked this information from the perspective of the intersected GI vs the PI within the testing subsets of the study areas as seen in Figs. S1, S2, S3 and S4 in the Supplementary Materials. The left panel (same as upper panel of Fig. 10) shows the distribution of the area (in log-scale) of predicted landslides against that of the ground truth (manual) landslides. The right panel shows the distribution of the area of predicted landslides that intersects within the reference ground truth landslides against that of the reference ground truth landslides. As we see in the figures, except for the Papua New Guinea region, the overall predicted landslide area within the ground truth landslides were pretty close to each other, almost representing the data points in a 45° line buffer zone. This shows that the most of the individual predicted landslide area were quite similar to the area of the individual landslides in the GI. However, the overall area of the PI was a bit off from the reference ground truth in the case of Papua New Guinea despite high accuracies in the traditional metrics.

## Discussion

### Advantages and limitations of PLANET HR images

The daily temporal resolution along with the global coverage are the biggest advantages that Planet Labs offers. Because the satellites have similar sensors, image pre-processing and analysis is streamlined and less uncertain. Since Planet images have a global coverage, expanding our approach to more regions for generating multi-temporal landslide inventories without much model re-training is feasible. The possibility of monitoring landslides at near-real time can also be envisioned because of the high temporal resolutions. However, the spatial resolution does present some challenges. Depending on the temporal window of investigation (i.e., whether before 2016 or after), we will have to employ either the RapidEye and PlanetScope images separately or collectively, and the spatial resolutions of 3-m and 5-m are not the best if we are to map very small landslides as they are often missed out. We remind here that the operational rule-of-thumb in raster image analysis states that the smallest detectable object is of dimension greater than 3 by 3 pixel, i.e. between 81 and 225 m^2^ in case of 3 and 5 m resolution. Furthermore, as hinted above, the prevalence of clouds, shadows, atmospheric noise, and image artefacts like haze makes it difficult at times to accurately and regularly map landslides. A possible solution to the problem of cloud coverage is to complement the optical image mapping with a parallel landslide detection procedure based on SAR imagery^54,55^.

### Automated pipeline using deep transfer learning as a mapping support

The automated approach is at present the only viable solution for mapping large areas at spatial and temporal accuracies suitable for scientific and operational purposes. However, robust, repeatable, and reliable procedures to automatize landslide detection over large data stacks of HR images are still non-existent or lacking. As a consequence, many landslide-affected areas remain unmapped because (1) they are difficult to map via traditional means, and (2) employing high-resolution images is expensive and labour-intensive, with a large part of the detection process still relying on Human Intelligence Tasks (HITs). Multi-temporal landslide inventories are fairly rare because spatiotemporal landslide mapping is not a trivial task and manual mapping is a considerably time-consuming process, whereas training semi-automated algorithm is challenging. Automated pipelines that seek to solve these concerns can therefore overcome these difficulties, significantly decreasing the need for HIT, and can be viable for the development of credible real-time monitoring and mapping of natural hazards at continental and global scales. In this study, we try to demonstrate that by training the model over a quite small percentage of each study area, we may thereafter correctly map the surrounding region (for 70,791 km^2^ in total).

Coupling Planet Lab images with deep transfer learning made our approach effective. It is very simple, easy, and feasible in mapping landslides in different regions. When moving to a new region to map landslides, the general approach would be to train the data from scratch to build a reliable/robust model, which would end up taking hours depending on the specifications of the machine, model architecture, data complexity, and data curation. However, with transfer learning, we are able to simply use the older weights and apply them directly on new training data for the newer region and the training essentially starts from where it was last left off. The transfer of knowledge and information from the previous domain makes it much easier and faster to train on a newer region, effectively reducing computational time by a significant amount. In terms of the detection of the landslides itself, the deep learning model is also capable of identifying landslides that are triggered by different factors. For example, landslides occurring in the later years of China and Nepal are monsoon-induced landslides. As long as the model can effectively “see” a trace of a landslide, it will pick them up and segment such traces as landslides. The capability of this deep transfer learning is also seen while predicting landslides in time. The deep transfer learning approach suggests a promising future for mapping landslides across time and space over much larger areas. Furthermore, the similarity of the FAD curves between the GI and PI is very promising, deeming the PIs to be very useful and close to the GIs while also portraying the applicability of the PIs for further use in the landslide community as well as in the broader geophysical community interested in the quantification and modelling of mass transport and its contribution to the global geological cycles.

### Factors influencing mapping accuracy

**Figure 11:**
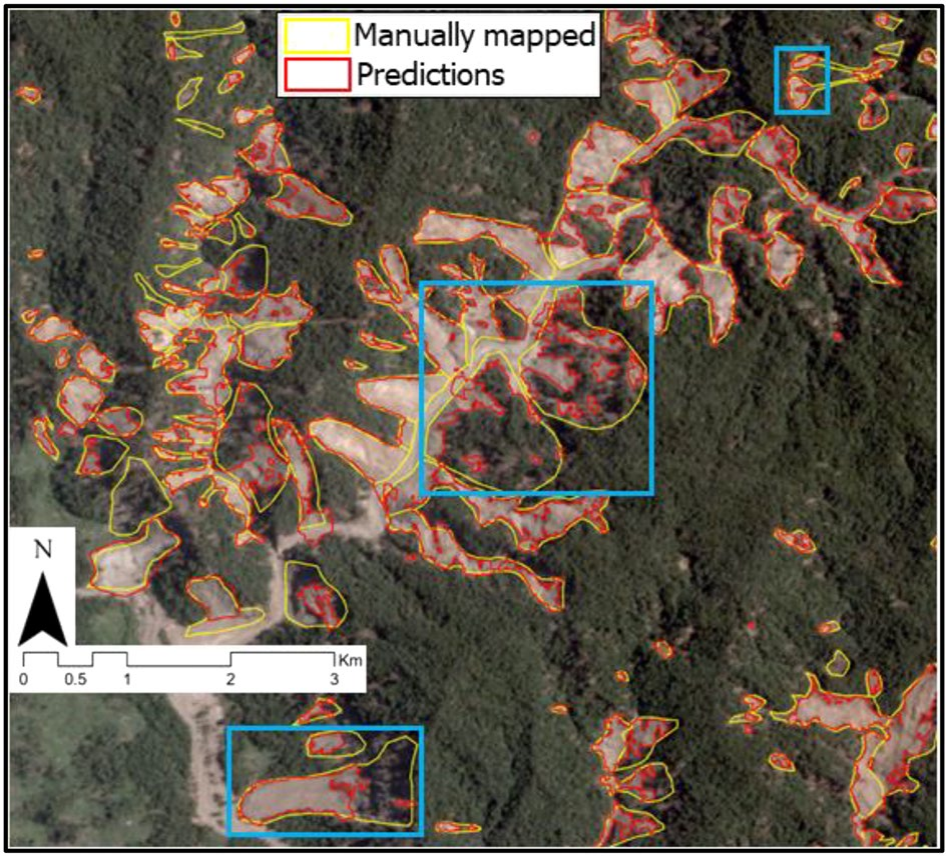
Example of landslides with radiometric signatures of vegetation, debris, and shadow in the co-seismic image of Papua New Guinea (Base image: PlanetScope, 2018). Blue boxes are areas of interest that depicts accurate predictions in the internal parts of the landslide body while missing out the head scarps.

Before analysing the temporal changes of the landslide geometries in the selected test subsets, it is essential to assess the achieved mapping accuracy and the possible error sources. Figures 10 and S1, S2, S3 and S4a) show that for all the cases except Papua New Guinea, the surface area of the predicted landslide are quite similar to that of the reference ground truth. The differences stem from the fact that modelled landslides suffer from geometric fragmentation which is persistent throughout all the study areas^56^. Therefore, landslide areas are often under-represented in the modelled outputs. We also notice that the largest deviations are observed in the co-seismic inventories. We attribute this to the following reasons: (1) model incapability in predicting the entire landslide body due to lack of training data, (2) satellite image artefacts like haze, shadows and cloud obscuration, (3) subjectivity and uncertainty in manually mapping landslides, which in turn may lead to (4) the detection of merely the “visible scars” in the terrain landscape without accounting for the portions of the landslide that are obscured by local disturbance factors such as for example, vegetation cover. Because of these phenomena, even if the model achieves high accuracy in the metrics utilized (Precision, Recall, and F1-scores), the model may still underpredict the landslide regions. As the Recall depicts the ability to effectively classify the landslide class, the model does a good job at identifying the landslide class while at the same time, often misses some pixels within the landslide body, thus leading to a lower value in the predicted area. Basically, it seems that the model performs adequately in highlighting where the landslides are but, sometimes, does not map the landslide polygon with the required accuracy. In Papua New Guinea, there are instances, for example in Fig. 11 (blue boxes), where internal parts of the landslides are detected but the entire head scarp is completely undetected. This could happen for example in rotational slides where the rigid-body movement failed to remove the vegetation cover at the head of the displaced mass with the consequence of leaving the spectral signature almost unchanged. The model would now simply predict the bare parts of the landslide body as landslides instead of the whole body. Prevalence of shadows also leads to incomplete mapping of the landslide bodies. We clearly see that the model is good at predicting landslides while avoiding the vegetation cover as seen in the figure. This can be explained by the fact that the model is trained with the NDVI and the added information coming from this band makes the model more efficient in avoiding vegetation signatures.

The presence of mountains in the region of New Zealand, notably in the north, north-eastern, and eastern regions of the study area, made it difficult for the model to predict landslides, as the mountains bore signals similar to bare soil and snow.

Uncertainties pertaining to the training data derived from inventories also have implications in affecting the mapping accuracy. Possible uncertainties comprise: (1) occurrence of landslides in the past but images were acquired later on due to unavailability (in case of China), (2) landslides might have occurred in different stages but we did not differentiate between the stages while curating and validating against the ground truth inventories, and (3) model inferencing was performed for the entirety of the study areas but testing data was only present within the testing windows for each area.

### Limitations of the automated pipeline

The quantity and quality of the training data play a vital role in the effectiveness of the deep learning model. A significant amount of training data is necessary for the neural network employed in this study. However, erroneous sampling strategy and therefore, incorrect labels can also potentially induce uncertainty and reduce the level of prediction capability of the model once we start upscaling both in space and time. The distribution of training data is crucial, as more training data for a given class may increase the method's sensitivity to that class. The model can learn features automatically, but since such models pertain a certain black-box nature, it is unknown what features and characteristics it learns of the target class^77^. We saw this in the varying values of the traditional metrics while evaluating the model performance for each year. A possible reason can be the variation of the landslide features over the years in the satellite images, which may increase the bias in the training data thereby leading to varying F1-scores.

The model's inability to predict landslides smaller than the manually mapped landslides is one of the reasons for the early halt of the fitted curve in the FAD. This can be due to the fact that expert manual interpretation allows to observe smaller landslides, which unfortunately, can be missed out by the model. Moreover, for some regions, the authors employed higher resolution remote sensing imagery to manually map the landslides and evidently, using sub-metre allows recognition of much smaller landslides compared to the 3-m and 5-m PlanetScope and RapidEye images, respectively. Such reasons can also influence the power-law divergence caused by under sampling smaller slides due to inadequate resolution of the remote sensing imagery^78^.

In comparison to manual delineation on images in the field, automatic mapping using remote sensing images is less accurate. However, we may determine that there is a significant degree of resemblance between the terrain factors obtained from manually delineated (i.e., ground truths) and automatically mapped landslides. Due to the ambiguities in remote sensing imagery, even manually delineated inventories requires validation. Therefore, in areas where the manual MT inventories are unavailable or out-of-date, we may use the outputs of automatic mapping for additional research or updating already-existing maps. Coupling manual interpretation and intervention with automated approaches can be a solution to further improve the detected landslides (in other words, the generated inventories).

### Future work

Combination of terrain information such as the elevation and slope steepness from DEMs can surely help in removing more of the FPs but this also needs to be experimented more carefully since it is not possible to acquire MT-DEMs easily. However, single date DEMs can also be experimented with as well to see the effect on FP elimination. Combination with other image sources like Sentinel-2 along with Planet images can also be an outlook to capture much of those landslides which are bigger in size. Exploiting the many bands that the former possesses, searching, locating and eliminating FPs along with capturing landslides in space and time could be a fruitful tactic.

To further improve the results, employing more complex and deeper network architectures can also be a strategy to better map landslides while also avoiding FPs and reducing FNs. The current model is built on top of the U-Net architecture however, using deeper encoders like the Res-Net to derive a ResU-Net^79^ architecture with multi-scale deep supervision attention network could better leverage the complexities of the data.

As we saw in Fig. 3, we can essentially differentiate the types of landslides based on its mechanism and cause. This information can be used to evaluate the spatial evolution (or distribution of activity) of the landslides in the study area with respect to their changing susceptibility levels over time^41,42^, evaluate the frequency of remobilised landslides, and possibly also comment on the legacy effects of the triggering events by looking at the landslide “recovery”^3,37^. The legacy effect based on the path of landslides has also demonstrated in the decay of landslide occurrences after the co-seismic event (as seen in Fig. 4), and this is caused by new or follow-up landslides overlapping with older landslides that causes greater susceptibilities in the region^45,80,81^. Additionally, by resorting to this approach, it is possible to study the slope dynamics concurrently using the outputs of the automated pipeline across vast regions, which might also offer crucial additional insight into their properties, impact to elements-at-risk, and the conditioning factors. We would also like to mention that our modelling approach does not cover the entire range of landslide typologies, i.e., we do not classify the landslide based on movement types as that is out-of-scope and is a completely different problem which is not addressed in this study. Another important topic where MT-inventories are beneficial is in the study of linking mass wasting from landslide occurrences into the channel networks which impacts the overall sediment budget of the channel system. According to recent studies, the increased landslide activity has increased the amount of material in river channels as a result of sediment transportation from failed slopes^82–85^. For such studies, the precedence for generating MT- inventories data sets becomes apparent and therefore, is quintessential in order to monitor and examine channel network sedimentary budgets at much larger scales (ideally at the continental scale). The development of long-term MT-inventories can be facilitated by leveraging the automated pipeline suggested in this study, and it can be the key to understanding such phenomena.

## Conclusion

Automated endeavours for landslide mapping have become numerous, however, a transferable and scalable solution has not been achieved yet. In this study, we propose to address this transferability and scalability with a Deep Transfer Learning approach coupled with HR Planet Lab images, hence making this model a first of its kind in the automated landslide detection topic. The predictive capability, both in terms of the traditional computer vision metrics and the inventory statistics, illustrates the usability and applicability over large areas, even globally, to generate annual landslide inventories. However, the model predictability still needs to be further explored to target specific issues to improve the predictive capability for more complex regions, such like in New Zealand or arid regions. Despite the limitations, on the whole, the results show that the method is able to automatically map landslides with an acceptable accuracy over large areas with a relatively small effort in terms of training, labelling, and other HITs. Furthermore, it is the first time that such a transferable model has been developed to map landslides at such large scales not just over geographically and geomorphologically different regions but also over time. For studies related to hazard modelling, early warning, and the analysis of temporal evolution of landslides and the related geomorphology, multi-temporal inventories are essential. Our method offers an important step to achieve this for the landslide community.

In order to encourage the reproducibility and repeatability of the analyses described in this study, we emphasize that we share our data and codes in a GitHub repository.

## Materials

### Collection of multi-temporal satellite images

Multi-temporal HR remote sensing images from Planet Labs^86^ were downloaded which comprised of four bands: Blue, Green, Red, and Near-Infrared or NIR. Images spanning from 2009 to 2021 over the different areas were downloaded which are used for mapping the landslides temporally (Table 5). The spatial resolution of the PlanetScope and RapidEye images were 3-m and 5-m, respectively. Comprehensive information on the images with acquisition dates for each of the study areas can be found in Table 5. The downloaded images cover the regions of Nepal (WGS 1984 UTM Zone 45 N), China (WGS 1984 UTM Zone 48 N), Papua New Guinea (WGS 1984 UTM Zone 54S), and New Zealand (WGS 1984 UTM Zone 59S). A total of 25 images (Nepal: 7, China: 8, Papua New Guinea: 4, New Zealand: 6) were used for training, testing, and inference purposes. We selected the best possible cloud and shadow-free images for the analyses. The images have been orthorectified and their product level is "Analytic SR," which denotes that surface reflectance is represented by pixel values using 16 bits of bit depth. We pre-processed the images from each year's collection by band extraction, sharpening, and mosaicking to create an image that covered the study regions. In the case of China, images for the pre-seismic years were not obtainable as the RapidEye satellite was launched only on August 2008.

**Table 5:** Information about the satellite images for each region.

Years	Acquisition dates	Pixel size (m)
**Gorkha, Nepal**		
Pre-seismic (2013)	07-11-2013	5
Pre-seismic (2014)	30-11-2014	5
Co-seismic (2015)	09-11-2015	5
Post-seismic (2016)	04-11-2016	3
Post-seismic (2017)	12-11-2017	3
Post-seismic (2018)	24-10-2018	3
Post-seismic (2019)	10-11-2019	3
**Wenchuan, China**		
Co-seismic (2009)	29-11-2009	5
Post-seismic (2011)	08-07-2011	5
Post-seismic (2013)	12-05-2013	5
Post-seismic (2014)	25-07-2014	5
Post-seismic (2017)	11-07-2017	3
Post-seismic (2018)	12-08-2018	3
Post-seismic (2019)	15-08-2019	3
Post-seismic (2021)	03-08-2021	3
**Porgera, Papua New Guinea**		
Pre-seismic (2017)	03-11-2017	3
Co-seismic (2018)	23-03-2018	3
Post-seismic (2019)	17-03-2019	3
Post-seismic (2020)	01-12-2019 and 22-01-2020	3
**Kaikōura, New Zealand**		
Pre-seismic (early 2016)	10-01-2016 and 24-02-2016	5
Co-seismic (late 2016)	01-11-2016 and 28-01-2017	3
Post-seismic (2018)	19-01-2018	3
Post-seismic (2019)	15-12-2018 and 25-01-2019	3
Post-seismic (2020)	11-11-2020	3
Post-seismic (2021)	31-12-2021	3

We did not employ a digital elevation model (DEM) in our study since the landscape of the failing slopes may change after an event, and acquiring MT-DEMs for each year using a global DEM is not practical. Moreover, in order to generalize the model at a bigger scale, it is important to employ data that can be easily obtained and does not require extensive pre-processing. In other words, we aimed to make the training process challenging for the model as we envision the detection of landslides at different spatiotemporal locations and varying scales. *NoData* is quite common in remote sensing images and is detrimental in the context of machine learning or deep learning as they reduce model efficiency as the model trains over time. As the *NoData* values do not contribute at all, spectrally speaking, these values can lead to gradient explosions whereby large error gradients accumulate during model training and result in delayed updates in the weights of the networks, thus making the model unstable and unable to learn from the training data^87^. Therefore, *NoData* sample data were removed in this regard.

### Collection of multi-temporal inventories

Apart from satellite remote sensing images, manually mapped landslide polygons are also crucial while training supervised models for image classification. For all the cases under consideration, the co-seismic landslide inventories are already available in the literature for the entire areas affected by landslides. However, MT inventories are only available for the Gorkha and Wenchuan cases (Table 6) and in the latter study area^66^, no inventories were generated for some of the years we examine (e.g., 2019 and 2021). Therefore, to fill those gaps in the gathered collection of MT inventories and also to address some minor edits/re-digitization in the existing inventories to cater for efficient training of the model, we manually mapped landslides for a subset of examined area in each case. Some of the inventories gathered from the literature (e.g., for Wenchuan^66^ and for Kaikōura^88^) were digitised using remote sensing imagery of different resolutions (such as SPOT, Worldview, orthophotos, and Sentinel-2), which could lead to the identification of landslides with different level of detail when compared to images from PlanetScope and RapidEye. Therefore, these minor edits would provide a better delineation of the landslide bodies for modelling purposes. Moreover, having a precise ground truth both for validation and test set allows for reliable calibration while model training and an accurate evaluation of model performances.

**Table 6:** Accessible datasets through the literature on the MT-inventory sources and triggering mechanism.

Location	Triggering event	Source
Gorkha, Nepal	Earthquake + Rainfall	^56^
Wenchuan, China	Earthquake + Rainfall	^72^
Porgera, Papua New Guinea	Earthquake	^3^
Kaikōura, New Zealand	Earthquake	^88^

## Methods

**Figure 12:**
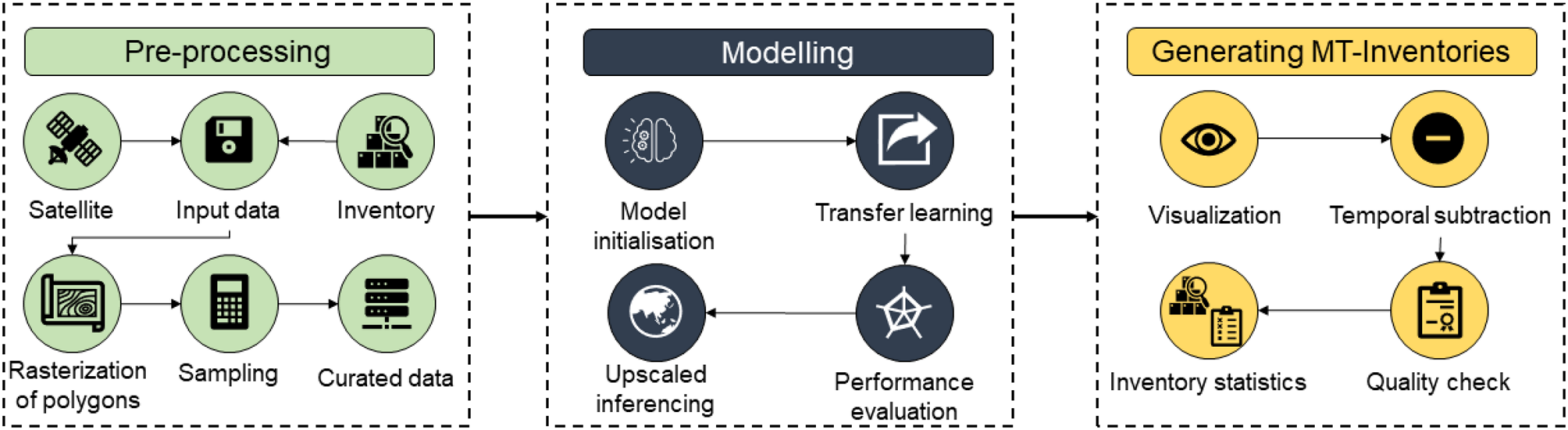
Conceptual framework of the automatic MT landslide mapping method based on deep transfer learning.

The framework depicted in Fig. 12 shows the approach taken to generate MT landslide inventories in different regions. Briefly, we first extracted several satellite images from Planet and developed orthorectified pixel-based composites of the images. To adopt a supervised classification routine, we sampled the curated data from the literature along with the satellite images into training and testing sets. Secondly, we initialised the training regime of the model and tested the model, both over space and time. We then performed transfer learning to re-train landslide instances in the other regions of interest while also evaluated the model for each temporal window. Upscaled inferencing was carried out to map landslides for the entirety of the study areas explicitly for each temporal window. Thirdly, we performed temporal subtraction to obtain the true MT inventories for each region. Finally, we performed a quality check of the inventories to shed more light on the statistics of each of the MT inventories for each region. All outputs of the model are binary images of values 0 (non-landslides) and 1 (landslides) where the 1 s are converted into geocoded landslide polygons using the GDAL and Shapely libraries in Python.

## Automatization of landslide mapping

### Preparation of training data

Training samples are key in supervised learning as the model learns based on the samples annotated as reliable ground truth by the user. Training samples consists of two parts: image patches and the corresponding masks of landslides. The former are patches of satellite images where we also calculated the NDVI for each of the MT images to acquire five-band/channel images for all study areas. NDVI products help differentiate landslide areas from non-landslide areas^89^ and therefore using this information helps to improve the classification of pixels for identifying the landslides. The latter are landslide polygons rasterized into images that acts as the labels for the respective satellite image patches. The training data for Gorkha are directly obtained from a previous study^56^ whereas in the Wenchuan and Kaikōura cases, we edited the landslide polygons as the landslide samples were not as precise as what our method requires. For example, we removed the landslide polygons if they were occurring under cloud cover and/or relief shadows to avoid errors in the understanding of the spectral and spatial configurations of the landslides as such samples can mislead the model into considering non-landslide pixels as landslide samples. Therefore, we amended such training samples catering toward efficiently training the deep learning model. In other scenarios, for example in Papua New Guinea, the obtained inventories^3^ were untouched despite some landslide samples had the presence of materials which consisted of radiometric signatures resembling that of vegetation on top of the landslides.

We employed data augmentation to the training set to increase the amount of training data and to improve the generalization of the modelling approach. Data augmentation, a strategy to enhance the amount and diversity of training data, is essential since it can improve the quality of our sparse training data. We also remove zero-valued patches (training patches with no landslide information) to mitigate the imbalance in the sample distribution between the positive (landslide) and negative (non-landslide) classes^90^. Flipping, blurring, sharpening, shearing, and rotating data augmentation techniques were utilised in this work since it results in the creation of new landslide patches with realistic orientation and spectral responses. We used the Python library *image* (https://imgaug.readthedocs.io/en/latest/) that implements these approaches. To be more precise, we used flipping (horizontal and vertical), blurring (gaussian blur: σ = 0 and 3), sharpening (α = 0 and 1), shear (with factors − 20 and 20), and rotating (45° and 90°) after conducting numerous experiments to verify the most useful combinations.

#### Model architecture and tuning

**Figure 13:**
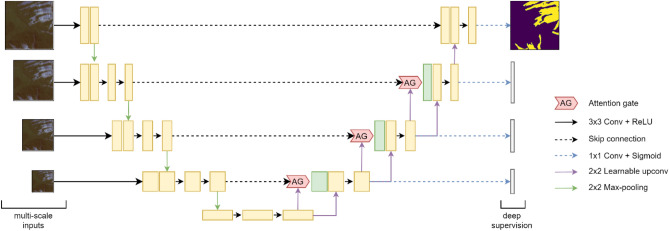
Model architecture of the Attention Deep Supervision Multi-Scale U-Net. (We generated TensorFlow implementation of the model which is shared with this paper in the Supplementary Material).

The U-Net network is a type of architecture used for image segmentation^91^, which involves separating an image into different parts or regions. It has been used in various tasks with good results^92^. The U-Net has a contracting path (encoder) that captures low-level image features such as landslide edges, boundary lines, textures, and an upsampling path (decoder) that captures high-level features such as shapes and patterns of landslides (steep slopes with vegetation cover, general landslide shapes w.r.t the landscape). The U-Net is good at producing accurate results even with a small amount of training data. However, it may have limitations when dealing with imbalanced data or small targets. To address these issues, we used a modified version of the U-Net called the Attention Deep Supervision Multi-Scale U-Net (ADSMS U-Net)^93^ (see Fig. 13). This model includes multi-scale inputs to gather feature information from both the target and the background at different scales, and soft attention gates to help detect relevant spatial information from low-level feature maps. We used the Adam solver^95^ for optimization, which adaptively learns and converges faster to reduce the loss. We trained and tested the ADSMS U-Net on a NVIDIA RTX 3060 GPU with 16 GB of RAM.

Hyper-parameter tuning is one of the most important steps in regulating the overall behaviour of the model. The goal is to find the optimum hyper-parameter combination that minimizes loss to deliver the best result. To avoid loss over-suppression, the ADSMS U-Net model controls each high-dimensional feature representation using Focal Tversky Loss, (Eq. 3) while the final output is controlled by the traditional Tversky Loss. This deep supervision technique^96^ necessitates semantically discriminative intermediate layers at all scales and also helps ensure that the attention unit has the ability to modify responses to a wide range of visual foreground material^93^. Traditionally modelling performance of (semi-) automated landslide mapping algorithms is checked by confusion matrix including true positives (TP), false negatives (FN), and false positives (FP) for each time period. TPs are correctly recognized landslides that are both geographically and temporally accurate, FNs are landslides that were missed out, and FPs are identified landslides that are not in the ground truth. All else can be considered as a true negative (TN), although this could be subject to a slightly larger uncertainty. The Tversky loss (Eq. 1) has the advantage of quickly adjusting and regulating the False Positives and False Negatives when using the α and β weights, thereby influencing model performance. By reducing the imbalance between the False Positives and False Negatives in consideration with the *landslide* and *non-landslide* classes, this parameter helps reduce model loss when training for increased accuracy.

1 $$ Tversky\,\, Loss = \frac{TP + \varepsilon }{{TP + \alpha \times FN + \beta \times FP + \varepsilon }} $$

where ε = A constant value of 0.0001 (by default) which prevents the loss from becoming infinite. α = Alpha parameter that adds weight to the FNs. β = Beta parameter that adds weight to the FPs.

The Focal Tversky Loss non-linearly focuses training on hard instances with a Tversky Similarity Index ($TI_{c}$) (Eq. 2) of less than 0.5, while excluding simple samples from the function.

2 $$ TI_{c} = \frac{{\mathop \sum \nolimits_{i = 1}^{N} p_{ic} g_{ic} + \epsilon }}{{\mathop \sum \nolimits_{i = 1}^{N} p_{ic} g_{ic} + \alpha \mathop \sum \nolimits_{i = 1}^{N} p_{{i\overline{c}}} g_{ic} + \beta \mathop \sum \nolimits_{i = 1}^{N} p_{ic} g_{{i\overline{c}}} + \epsilon }} $$

where $$ p_{ic}$$ denotes the probability of a pixel belonging to the landside class *c* while $$p_{{i\overline{c}}}$$ denotes the likelihood that a pixel belongs to the non-landslide class *c*. The same may be said of $$ g_{ic}$$ (ground truth landslide class) and $$g_{{i\overline{c}}}$$ (ground truth non-landslide class), respectively. In the case of a substantial class imbalance, hyperparameters can be modified to increase recall. Finally, Focal Tversky Loss (FTL_C_) function can be defined as:

3 $$ FTL_{c} = \mathop \sum \limits_{c} (1 - TI_{c} )^{{{\raise0.7ex\hbox{$1$} \!\mathord{\left/ {\vphantom {1 \gamma }}\right.\kern-0pt} \!\lower0.7ex\hbox{$\gamma $}}}} $$

where $\gamma$ ranges between 1 and 3.

### Transfer learning approach

Transfer learning aids in the optimization of model performance on a restricted number of samples because sufficient training data is difficult to get in real-world circumstances. Transfer learning is classified as cross-domain or cross-modal, depending on the target and source data domains^97^. In this work, cross-modal transfer learning was utilized, which is a method of exploiting pre-training models already trained on detecting landslides. Learning about edge, form, and texture detections from earlier “experiences”, then re-training the model on newer regions, makes the process of detecting landslides much more efficient and strongly reduces training time^98^. Naturally, this is done by freezing the initial layers of the model that has already learned the most simple features (e.g., edges, forms, and textures). Training on top of these frozen layers allows learning new deeper features about the landslides from different regions without losing previously acquired detection capabilities. Therefore, when the model is tasked on newer regions where the radiometric returns will be different for probably the same types of landslides, the transfer learnt model will adapt on top of these “known” features, thus saving computational time as well as detecting landslides on the new regions more efficiently by increasing dataset diversity. Basically, by training a model pre-trained in area A on landslides of area B, we aim at improving the generalization capability while at the same time keeping overfitting as low as possible.

**Figure 14:**
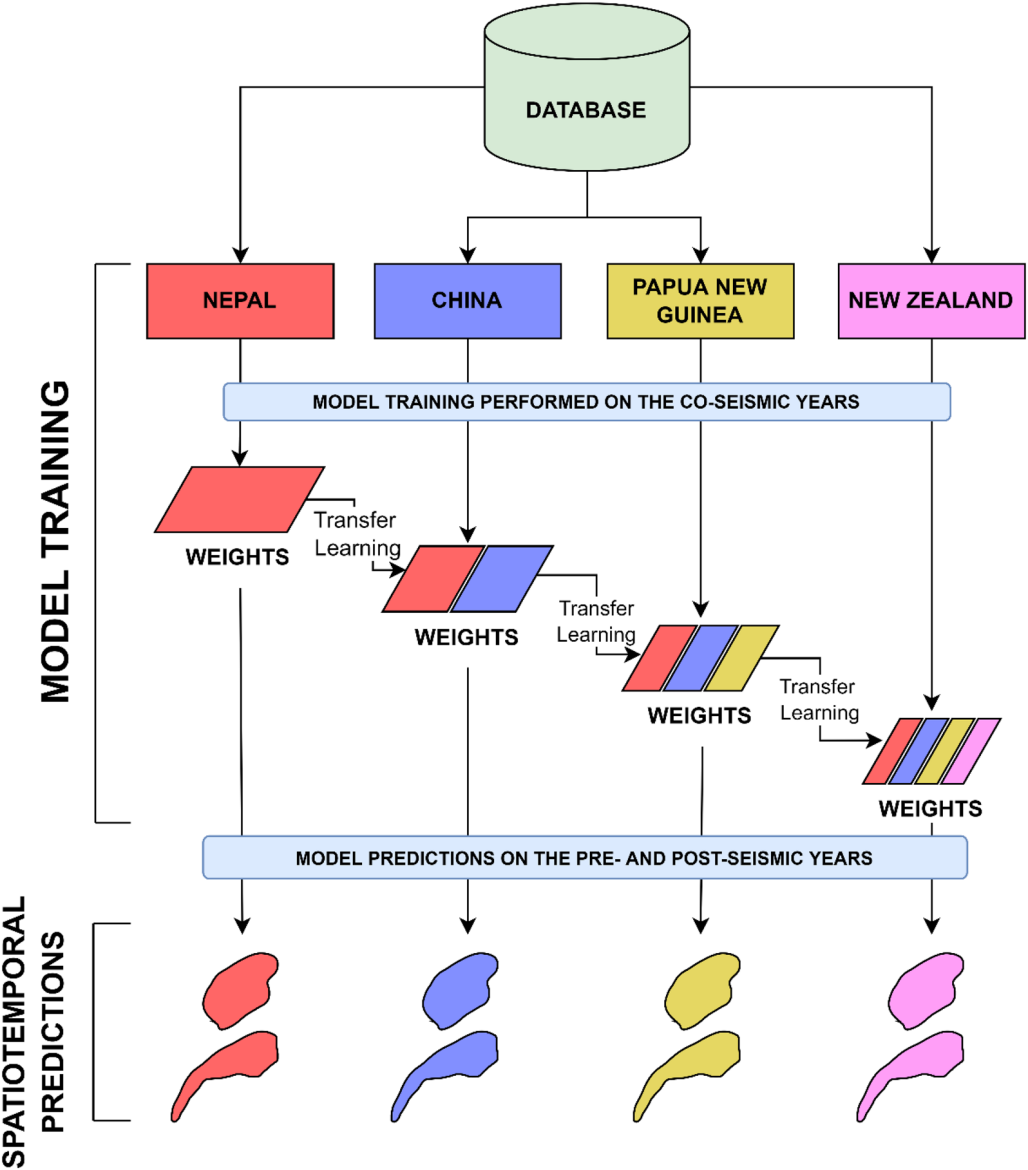
Schematic diagram of the transfer learning approach undertaken in this study. Weights are obtained from the first study area and then used along with training samples in the second study area for assistance while training in the second area. This is followed until the last study area where the model is trained using weights from the first three areas and samples of the fourth, respectively. An additional advantage of this modelling layout is that new study areas can be added at any moment producing a possible further model improvement in accuracy, generalization, and overfitting.

To this end, we developed a sequential tiered-based transfer learning approach to detect landslides on each study area, that exploits previous knowledge in a cascading sequence (Fig. 14).

To explain the transfer learning strategy holistically, let us consider the following:

R = Region.

R_T_ = Co-seismic year of the region (e.g., R_T′_: Region one; R_T″_: Region two, etc.)

R_T−N_ = Pre-seismic years.

R_T+N_ = Post-seismic years.

In the initial stage, we first train the deep learning model on the co-seismic year of the first region, R_T_. This is because of two particular reasons: (a) the event of R_T_ is the most interesting period as the shocks of the earthquake lead to the failure of hillslopes, and (b) R_T_ would also contain the greatest number of landslide occurrences and naturally, it is the most important period to map and assess post-event damage. Although we consider co-seismic events in this study, there is no impediment in using rainfall events to utilise this strategy. After training of the model, we test the model in time over the pre- (R_T−1_, R_T−2_…R_T−N_) and post-seismic (R_T+1_, R_T+2_…R_T+N_) years. In the second stage, we perform *transfer learning* by taking the trained weights obtained from R_T_ and train on a second co-seismic region, R_T′_. Therefore, R_T′_ is now trained with the knowledge of landslides from R_T_, giving us an updated model. We test this updated model over the pre- (R_T′−1_, R_T′−2_ …. R_T′−N_) and post-seismic (R_T′+1_, R_T′+2_ …. R_T′+N_) years to evaluate the performance of the model. We repeat the same strategy for regions R_T″_ and R_T′′′_. Through this method, we apply a tiered-based transfer learning approach on our deep learning model that effectively identifies landslides from different regions (i.e., the space) and over different temporal windows (i.e., the time). After attaining the trained weights, we use them to inference (or predict) on the entirety of each study area and time to obtain the respective predicted MT landslide inventories.

### Temporal subtraction and removal of erroneous polygons

**Figure 15:**
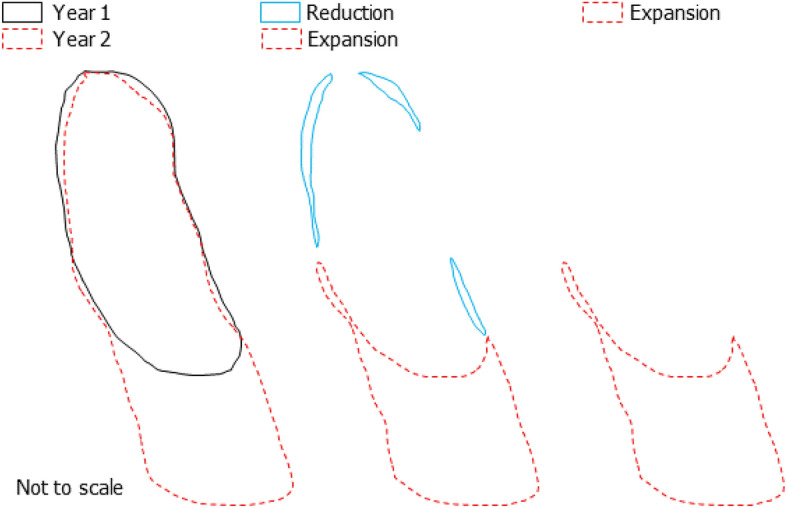
Example of landslide polygon subtraction. The first panel shows the boundaries of the landslides in Year 1 and Year 2, second panel shows the expansion and reduction part of the landslide after subtraction, and the third panel shows the expanded part after removal of the reduction parts.

Since the deep learning model predicts all the landslides that it could find in each image, the model would also detect landslides induced by past events. Therefore, in order to generate true MT inventories, we remove the older landslides from the map by using a post-processing algorithm that applies a temporal subtraction between years T and T−1 (where T is the base year). To accomplish the subtraction in Eq. (4), we utilized the python library Shapely (https://pypi.org/project/Shapely) and its "difference" function. In Shapely, the expanding areas and/or reducing areas were shown as geometries of the "Polygon" or "multiPolygon" type.

4 $$ E_{T} = P_{T} - P_{T - 1} $$

where E_T_ is the areal difference that, in case has positive value, may represent the areal expansion of the landslide in the time interval between T and T−1. In cases of reduction in areas (Fig. 15) caused by delineation errors, the “difference” operation would attribute towards generation of such narrow artefacts. We removed these parts after temporal subtraction by considering a buffer around the obtained expanded and/or new landslides to accurately calculate the landslide polygon attributes (perimeter and area).

Note that all post-event images are used to detect reactivated landslides and newly formed ones. We do not separate the expanded landslides from that of the original landslide body as *remobilised/reactivated landslides* and treat them as the same for model testing purposes.

### Accuracy assessment

We identified spatially independent subsets to assess the accuracy of the model. The subsets were designed so as to be representative of each area. The model predictions are assessed against the manually generated ground truth using the PlanetScope and RapidEye images (except for the co-seismic year since the co-seismic inventories were used from the literature) for each of the multi-temporal years for each study area.

The mapping accuracy of the applied method was calculated using the three accuracy metrics applicable for pixel-based segmentation algorithms. Precision (Eq. 5) here refers to the proportion of areas that were correctly classified as landslide areas. Recall (also known as sensitivity) is the amount of times landslide areas were classified accurately (Eq. 6), and F1-score (Eq. 7) is the weighted average of the precision and the recall that is used as a balance between the precision and recall.

5 $$ Precision = \frac{TP}{{TP + FP}} $$

6 $$ Recall = \frac{TP}{{TP + FN}} $$

7 $$ F1{-}score = 2 \times \frac{Precision \times Recall}{{Precision + Recall}} $$

To attempt an interpretation of mapping accuracies, as well as to be able to gain insights into the nature of the automatically generated multi-temporal inventories, we also studied the frequency-size statistics of both Ground Truth Inventories (GI) and Predicted Inventories (PI). Frequency–area distribution (FAD) curves typically depict landslide areas against the corresponding cumulative or non-cumulative landslide frequencies, and are often used to characterize the statistical properties of a landslide inventory. FAD trend of most landslide inventories diverges from a power law for small landslides. Slope of the power law (i.e., power-law exponent) is used to gain insight to the characteristics of the landslide size distribution and the volume of material that had failed^99^. The rollover indicates the region of the distribution where the slope of the distribution changes sign^100^.

In our study, we employ a probability density function (pdf) as a three parameter Double Pareto Simplified (DPS) function^101^, written as:

8 $$ p\left( {A_{L} } \right) = pdf{(}X{|}\alpha , \beta , t) = \left[ {\frac{{ \beta \left( {t^{\alpha } } \right)}}{{\left( {1 + \left( \frac{x}{t} \right)^{ - \alpha } } \right)^{{\left( {1 + \left( {\frac{\beta }{\alpha }} \right)} \right)}} x^{{\left( {\alpha + 1} \right)}} }}} \right]{ } $$

here *A*_*L*_ stands for the area of the landslide, α is the scaling exponent that chiefly regulates the power function for large sizes, *β* is the scaling exponent that controls the power function for small sizes, and *t* constrains the rollover position^102^. We employ the Double Pareto simplified fit to the FAD^103^ for the co-seismic years by tallying our predictions against the reference ground truth for the entirety of the four study areas (not the spatially independent subsets) found in the literature in order to understand the similarities and/or differences between the inventories.

**Figure 16:**
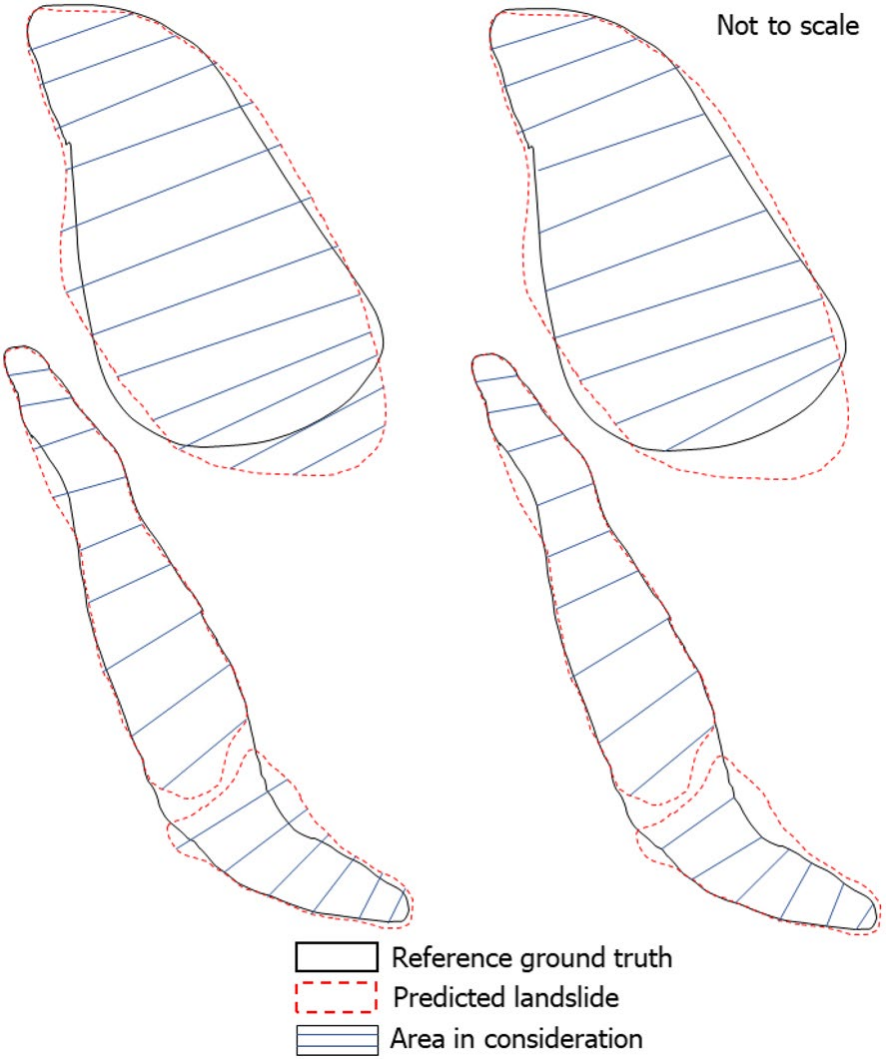
Example of the predicted landslide area versus the reference landslide area (left) and predicted landslide area within the reference landslide body versus the reference landslide area (right).

One of the caveats with the traditional methods also mentioned above is that they do not provide information regarding the accuracy of individual landslide polygon. For instance, Eqs. (5–7) depict how many of the landslide pixels were correctly recognised by the model and reports solely on the premise of spatial location and extent. FAD curves, on the other hand, show if overall size distributions are matching. However, the area of individual landslides is also important and it needs to be considered in terms of how well the model detects the overall landslide body as an object. Often, models will detect two or multiple landslide objects for one reference landslide (ground truth) due to reasons like: (1) obstruction in the continuation of a landslide body by vegetation or debris as seen in a satellite image, (2) incapability of the model to detect all the landslide pixels. This leads to fragmented detections^56^ of the landslide body (Fig. 16). However, in the current literature, this part of the problem is not discussed in terms of how well the *landslide areas* are predicted with reference to the actual landslides. The area of landslides is very important as it is always used in the succeeding phases of landslide hazard and risk assessment^101,104–106^ but the performance of automated inventories based on the *area* has not been evaluated, despite many literature discussing the usability of machine/deep learning algorithm derived inventories. Therefore, we report (a) the area of each predicted landslide versus the reference ground truth, and (b) the intersected area of each predicted landslide within the reference ground truth against the reference ground truth (refer to Fig. 16). We do this by assigning identification codes of each *reference ground truth landslide* to that of the *predicted landslide* when/where the latter intersects the former.

Landslide statistics were also evaluated for the manually annotated GI and PI over the different years using information such as total number of landslides (T_L_), total landslide area (T A_L_), maximum (Max A_L_) and minimum landslide area (Min A_L_). Reminder that the ground truth used for comparison are within the testing windows for each study area (not the entire study area). Tables S1, S2, S3, and S4 in the Supplementary Materials portrays the results of these landslide statistics.

## Supplementary Information

Supplementary Information.

## Data Availability

In order to make our study as transparent and reproducible as possible, we share the necessary data and codes to replicate our findings. We share the satellite images and also the predicted multi-temporal inventories in a polygon shapefile for further experimentation purposes. We encourage interested users/researchers to try out the models for their use cases. The link to the GitHub repository: https://github.com/kushanavbhuyan/Large-scale-multi-spatiotemporal-landslide-mapping.
