# Supplementary material for: Generating multi-temporal landslide inventories through a general deep transfer learning strategy using HR EO data
Source: Sci Rep. 2023 Jan 4;13:162. doi: 10.1038/s41598-022-27352-y (PMC9813262; doi:10.1038/s41598-022-27352-y)
Supplement: Supplementary file 1 — Supplementary Information. [file 41598_2022_27352_MOESM1_ESM.pdf]

Supplementary Materials

Title: Generating multi-temporal landslide inventories through a general deep transfer learning strategy using HR EO data

Authors:
Kushanav Bhuyan [1,2*], Hakan Tanyaş [2], Lorenzo Nava [1], Silvia Puliero [1], Sansar Raj Meena [1,2], Mario Floris [1], Cees Van Westen [2], Filippo Catani [1]

[1] Machine Intelligence and Slope Stability Laboratory, Department of Geosciences, University of Padova, 35131 Padua, Italy

[2] Department of Applied Earth Sciences, Faculty of Geoinformation Science and Earth Observation (ITC), University of Twente, 7514 AE Enschede, The Netherlands

Correspondence: kushanav.bhuyan@studenti.unipd.it

Table S1: Comparison of landslide statistics for the ground truth inventories (within testing windows) versus the predicted inventories in Nepal.

Year	Ground Truth Landslide Inventory (GI)				Predicted Landslide Inventory (PI)			
	Total number of landslides (T_L)	Landslide area (in m²)			Total number of landslides (T_L)	Landslide area (in m²)		
		Total (T A_L)	Minimum (Min A_L)	Maximum (Max A_L)		Total (T A_L)	Minimum (Min A_L)	Maximum (Max A_L)
2013	31	513,304.8	216.3	148,948.1	34	448,171.0	250.2	186,958.0
2014	10	47,019.2	824.3	20,810.8	21	59,788.7	253.2	20,810.8
2015	95	1,031,443.9	303.0	115,606.0	109	978,658.1	263.7	205,380.2
2016	19	74,930.2	865.1	11,397.0	28	60,344.3	430.0	9,359.3
2017	9	83,192.3	599.1	46,386.7	18	63,188.5	267.6	29,694.1
2018	3	9,513.1	1,439.3	4,428.4	12	13,910.2	314.0	4,150.7
2019	4	49,150.7	2,080.5	23,420.5	24	87,207.3	359.2	20,308.7

Table S2: Comparison of landslide statistics for the ground truth inventories (within testing windows) versus the predicted inventories in China.

Year	Ground Truth Landslide Inventory (GI)				Predicted Landslide Inventory (PI)			
	Total number of landslides (T_L)	Landslide area (in m²)			Total number of landslides (T_L)	Landslide area (in m²)		
		Total (T A_L)	Minimum (Min A_L)	Maximum (Max A_L)		Total (T A_L)	Minimum (Min A_L)	Maximum (Max A_L)
2009	2353	11,701,798.3	250.0	750,542.4	2100	15,463,307.7	275	902,525
2011	194	578,235.4	254.0	42,608.0	332	753,555.9	300.1	37,563.6
2013	5	14,329.0	649.5	7,009.8	20	20,817.5	257.2	5,237.8
2014	161	829,995.3	258.6	202,374.2	140	1,015,578.5	263.1	205,548.1

2017	8	51,442.6	1,319.8	21,230.6	10	48,716.6	334.6	20,025.0
2018	35	330,319.8	312.7	81,465.8	29	314,944.0	269.2	102,840.8
2019	42	157,279.7	279.7	49,514.5	42	120,489.3	282.6	49,247.3
2021	38	52,777.8	492.0	14,403.3	42	56,228.4	301.1	10,080.0

Table S3: Comparison of landslide statistics for the ground truth inventories (within testing windows) versus the predicted inventories in Papua New Guinea.

**Year**	**Ground Truth Landslide Inventory (GI)**				**Predicted Landslide Inventory (PI)**			
	Total number of landslides (T_L)	Landslide area (in m²)			Total number of landslides (T_L)	Landslide area (in m²)		
		Total (T A_L)	Minimum (Min A_L)	Maximum (Max A_L)		Total (T A_L)	Minimum (Min A_L)	Maximum (Max A_L)
2017	67	126,458.1	123.5	10,303.7	74	142,513.3	373.6	16,369.1
2018	1143	9,755,998.9	131.9	448,218.7	1108	8,182,051	252	632,241
2019	40	203,635.9	369.7	30,997.7	65	220,381.4	231.6	24,424.0
2020	5	6,552.2	155.1	3,136.2	5	8,123.2	314.4	2,394.7

Table S4: Comparison of landslide statistics for the ground truth inventories (within testing windows) versus the predicted inventories in New Zealand.

**Year**	**Ground Truth Landslide Inventory (GI)**				**Predicted Landslide Inventory (PI)**			
	Total number of landslides (T_L)	Landslide area (in m²)			Total number of landslides (T_L)	Landslide area (in m²)		
		Total (T A_L)	Minimum (Min A_L)	Maximum (Max A_L)		Total (T A_L)	Minimum (Min A_L)	Maximum (Max A_L)
2016	43	487,445.7	718.3	138,233.2	58	588,852.7	273.4	138,838.1
2017	246	3,506,832.6	676.7	165,943.8	357	3,701,073.8	251.8	108,819.1
2018	14	51,060.0	1,232.8	6,948.1	29	47,331.1	274.8	5,804.4
2019	7	42,311.3	618.4	24,353.6	6	62,441.7	1,721.6	35,343.5
2020	4	24,433.6	3,418.3	11,382.4	6	45,991.9	3,052.0	14,414.4
2021	3	47,301.3	1,426.0	38,263.0	4	69,507.7	7,454.6	32,307.9

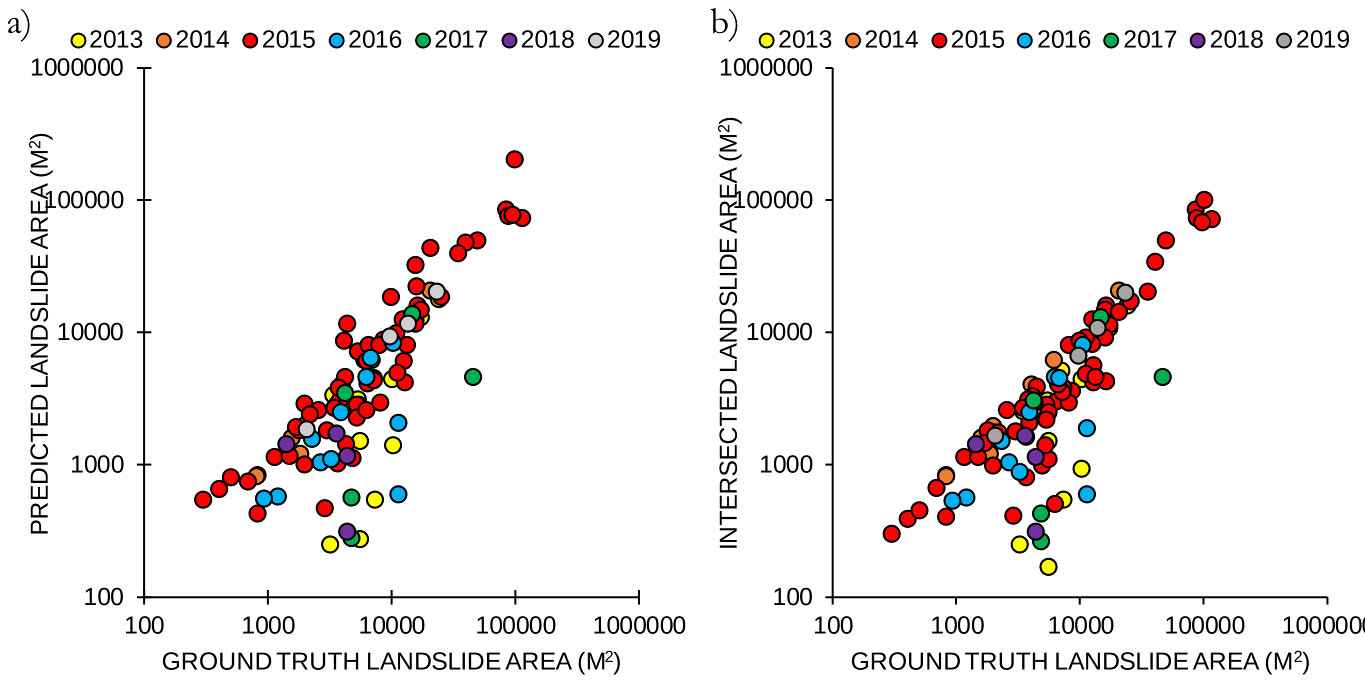

Figure S1: NEPAL - Difference in area between the predicted landslides versus ground truth landslides (a). Difference in area between the predicted landslides inside the ground truth versus ground truth landslides (b).

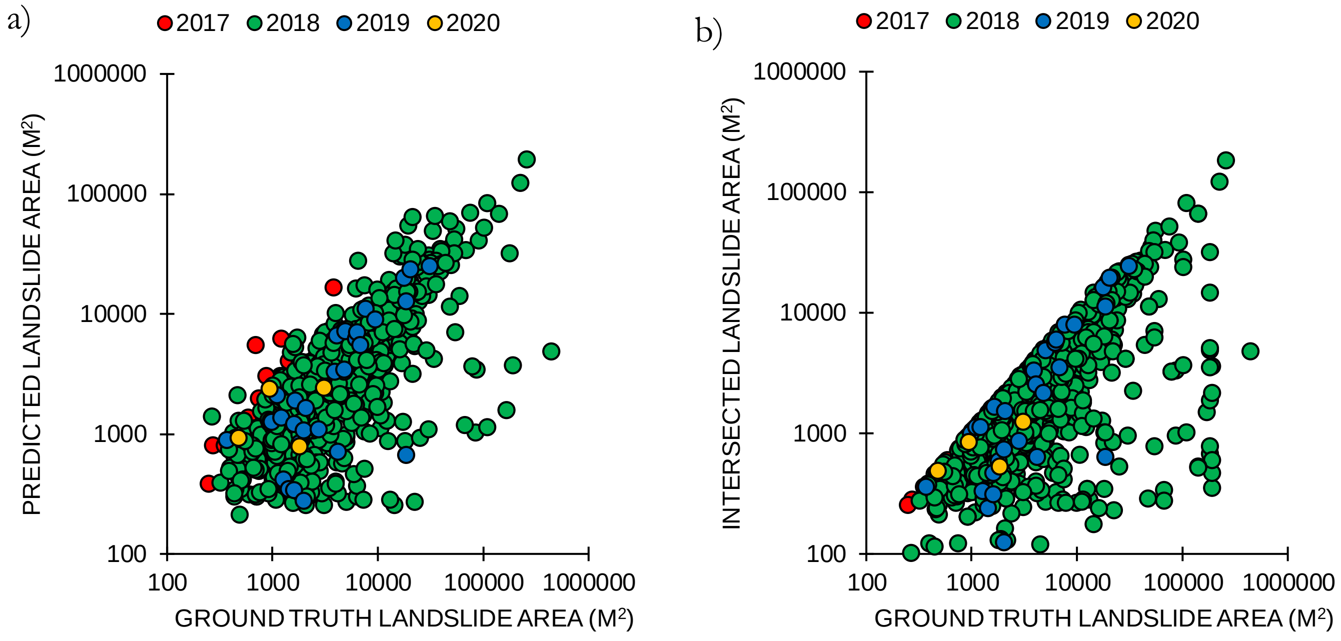

Figure S2: PAPUA NEW GUINEA - Difference in area between the predicted landslides versus ground truth landslides (a). Difference in area between the predicted landslides inside the ground truth versus ground truth landslides (b).

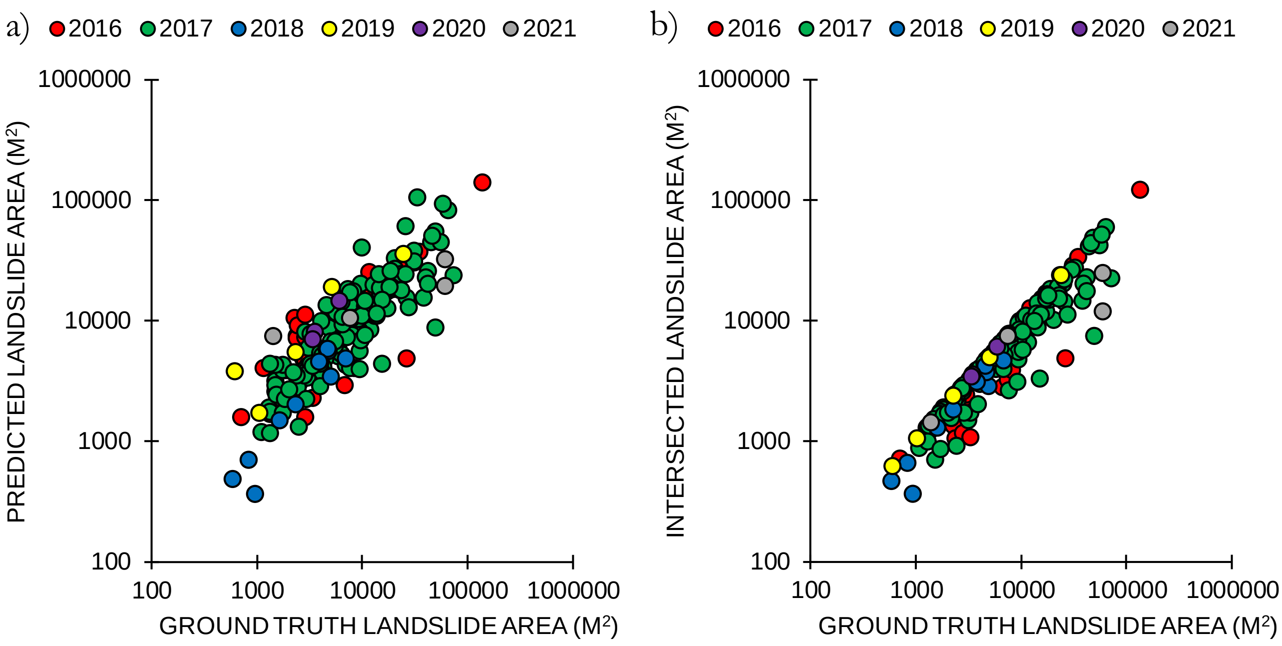

Figure S3: NEW ZEALAND - Difference in area between the predicted landslides versus ground truth landslides (a). Difference in area between the predicted landslides inside the ground truth versus ground truth landslides (b).

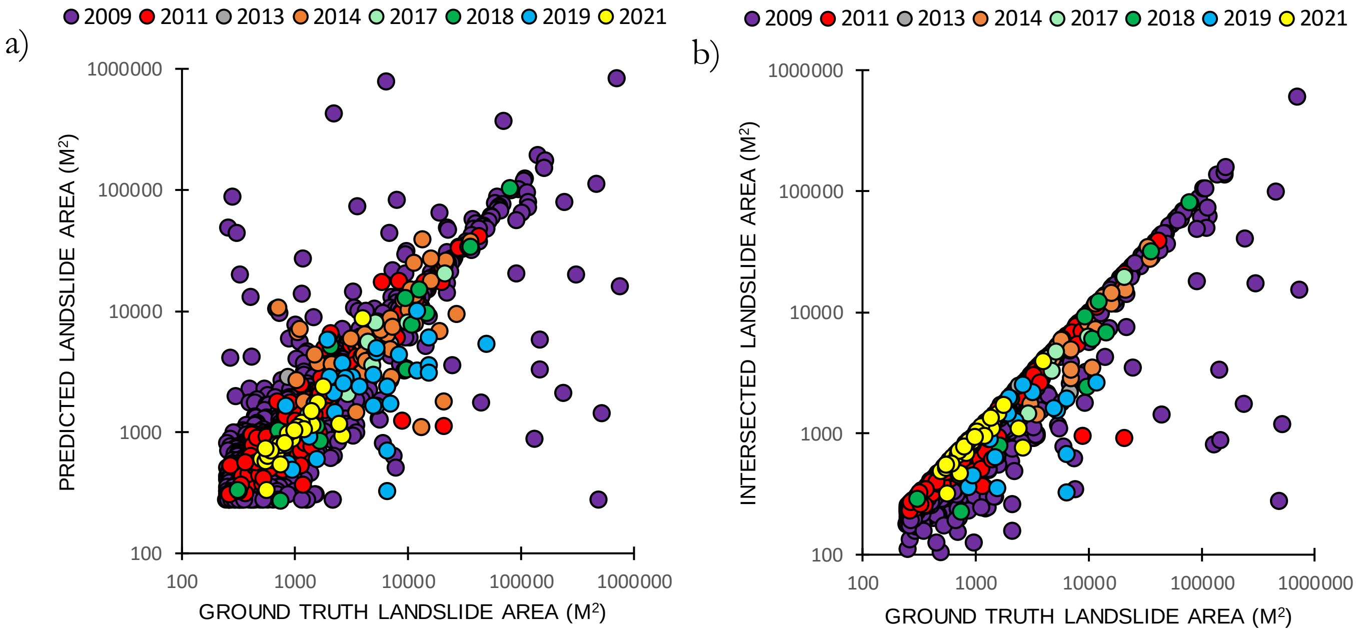

Figure S4: CHINA - Difference in area between the predicted landslides versus ground truth landslides (a). Difference in area between the predicted landslides inside the ground truth versus ground truth landslides (b).

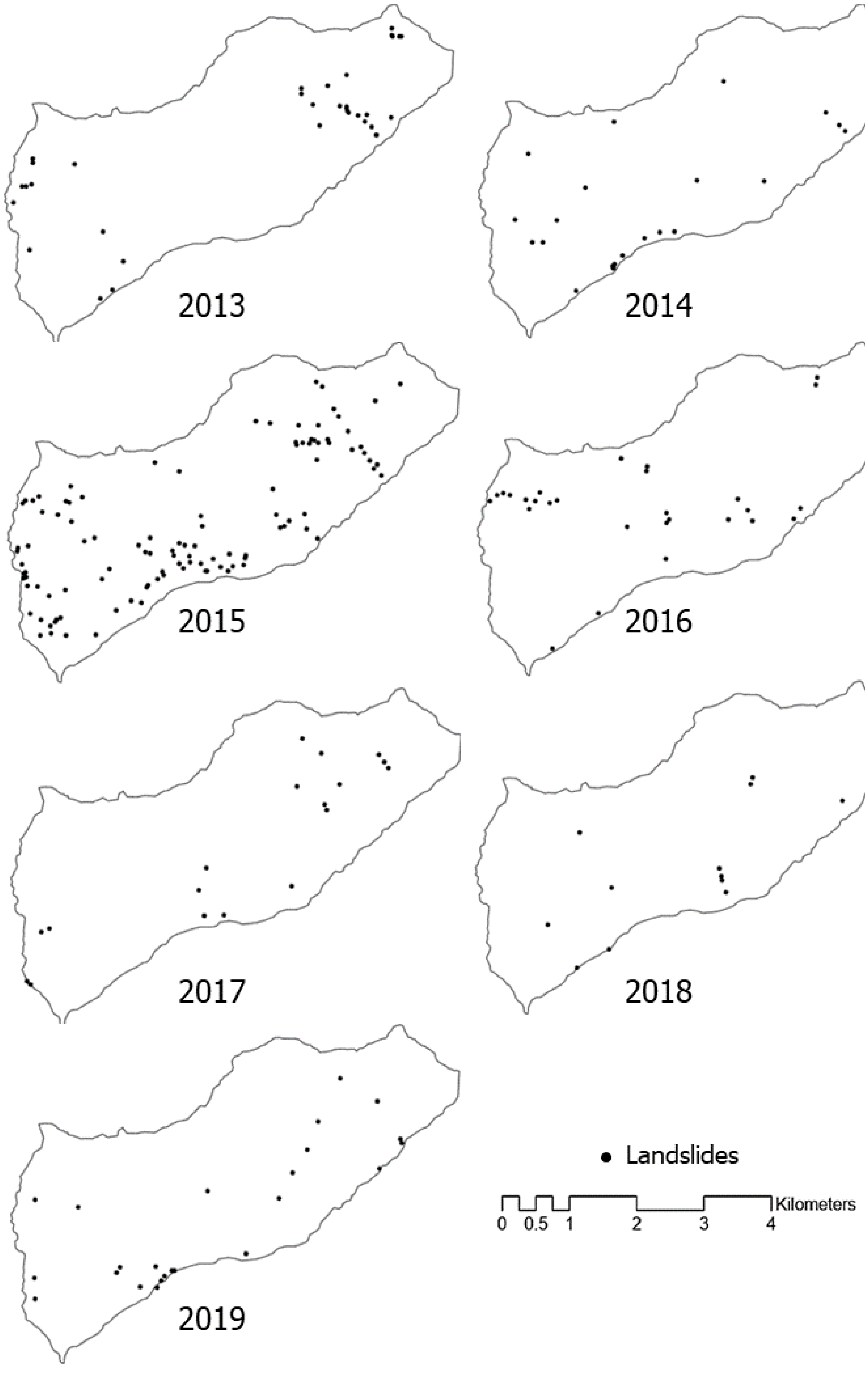

2013
2014
2015
2016
2017
2018
2019
Landslides
0
0.5
1
2
3
4
Kilometers

Figure S5: The temporal evolution of landslides in Rasuwa, Nepal from 2013 till 2019. (Note: For the sake of visibility, we presented landslides as points although they were originally mapped as polygons.)

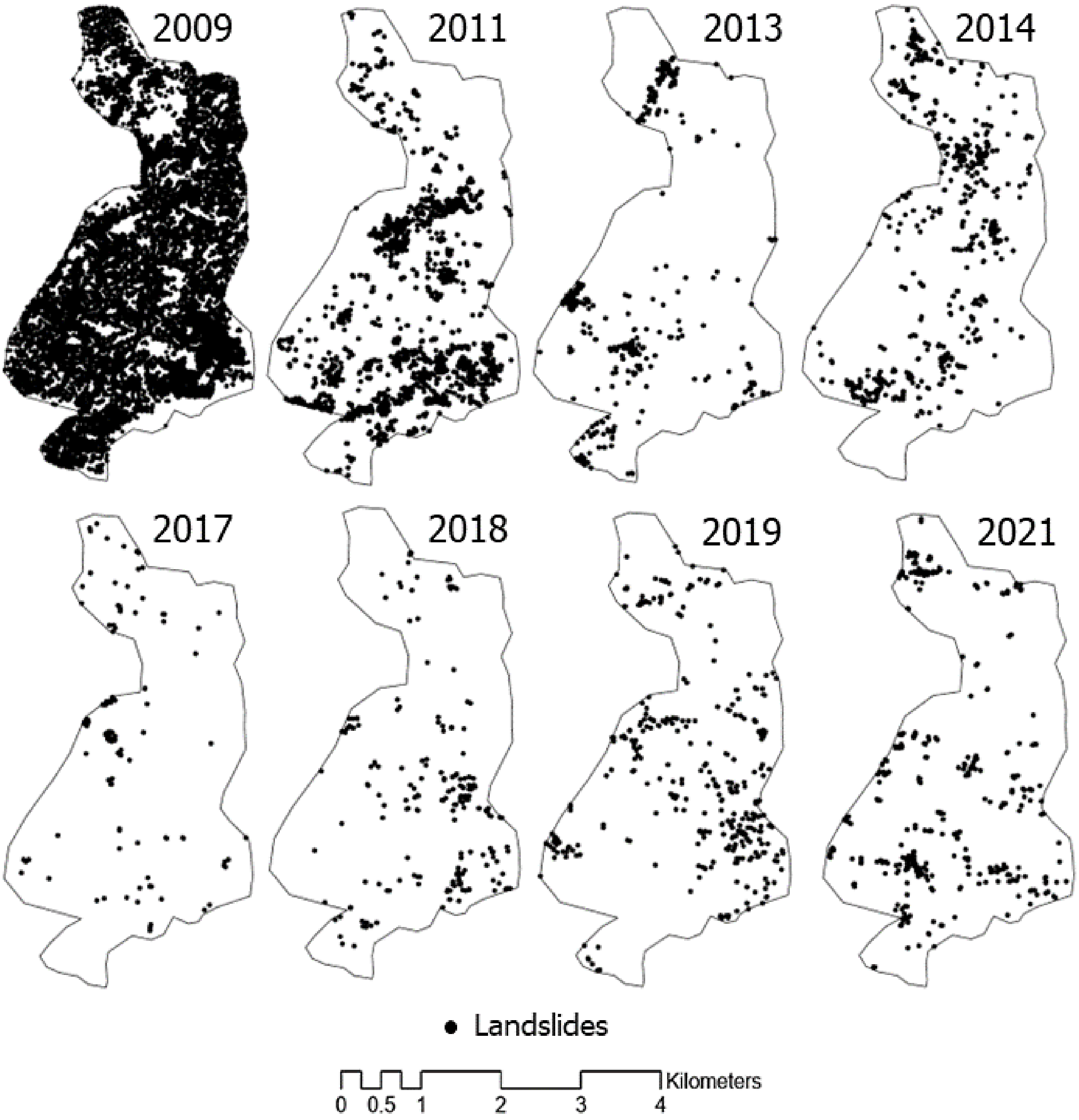

Figure S6: The temporal evolution of landslides in Wenchuan, China from 2009 till 2021. (Note: For the sake of visibility, we presented landslides as points although they were originally mapped as polygons.)

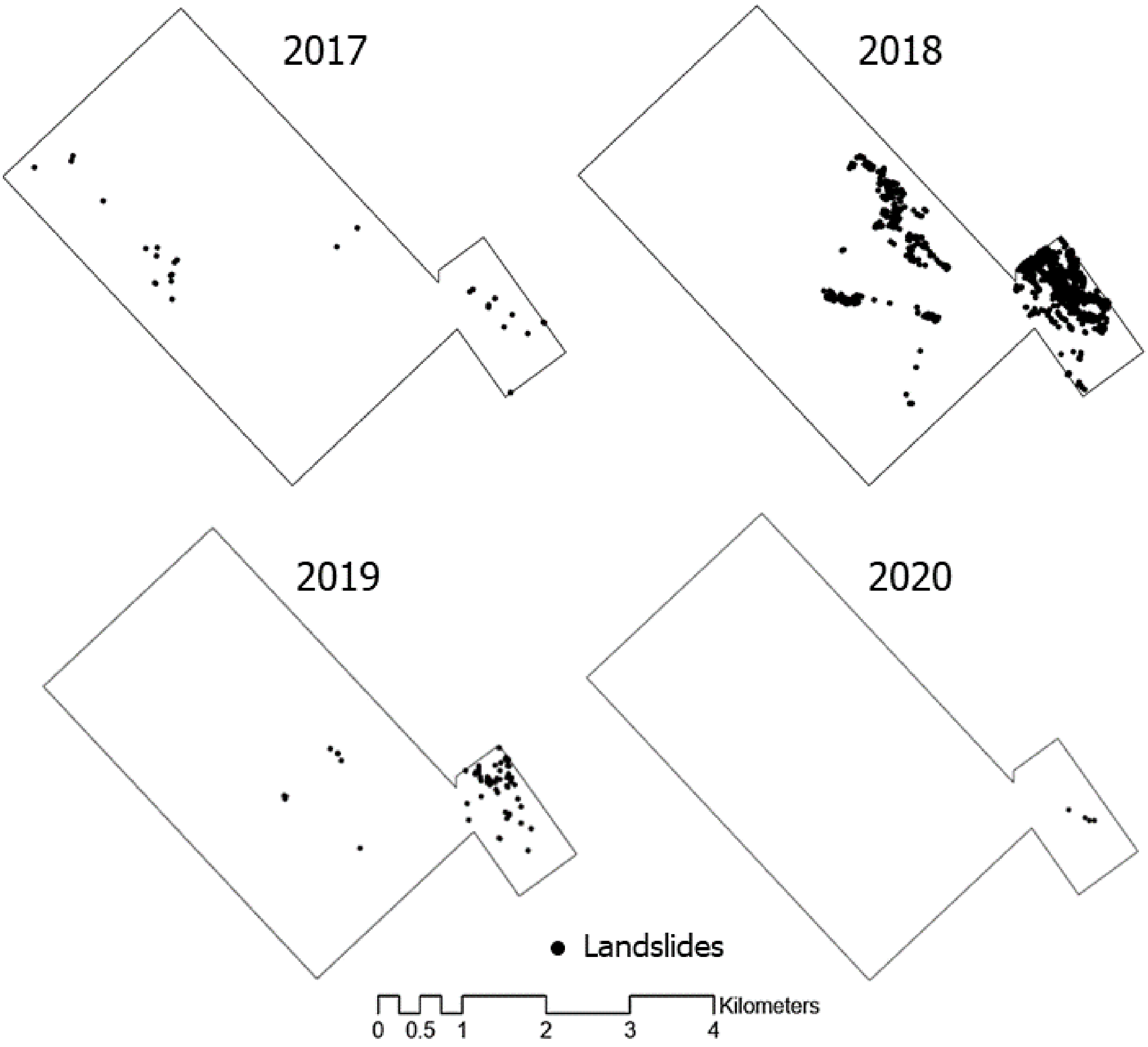

Figure S7: The temporal evolution of landslides in Porgera, Papua New Guinea from 2017 till 2020.

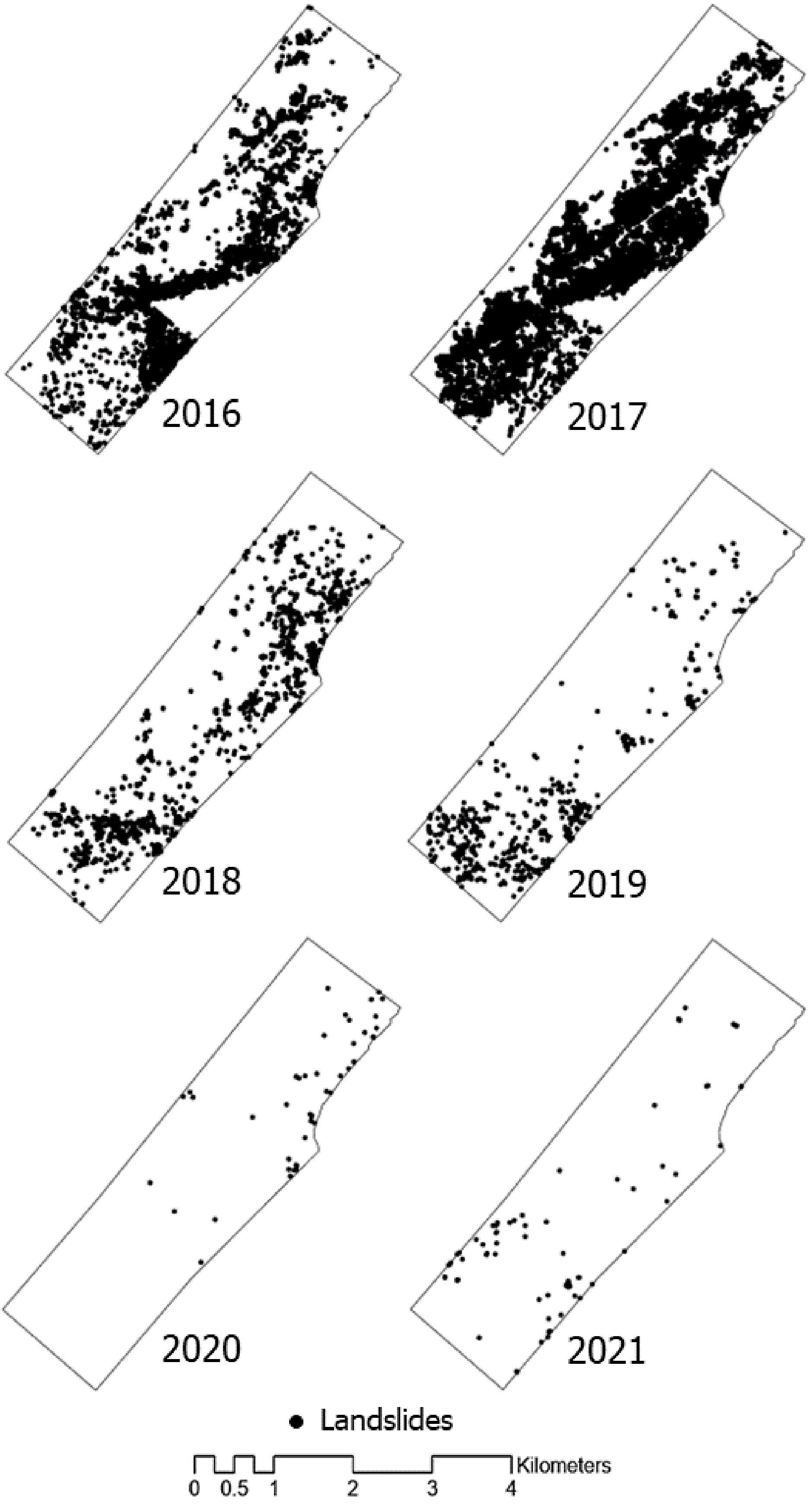

Figure S8: The temporal evolution of landslides in Kaikōura, New Zealand from 2016 till 2021.

Model architecture

We provide TensorFlow implementation of the model shared in the following paragraph. We adopt the encoder-decoder structure built using the ADSMS U-Net model while following the naming convention used in the GitHub repository of Johnson, Alahi, and Fei-Fei (2016).

Here, let, *k* denote the number of filters, *t* denote the concatenation operation, *s* denote the strides, and *max* denote a 2 x 2 max pooling layer. *UP* denotes a 3 x 3 upsampling convolution operation with *k* filters and *s* strides. *AP* denote Average Pooling, where average pooling operation is performed to obtain a multi-scale input image pyramid for better intermediate feature representations. {*c3s1tmax – k*} denote a 3 x 3 Convolution-ReLU layer with *k* filters, stride 1, and concatenation with the respective convolution input layer and a max pooling layer. {*c3s1B – k*} denotes a Convolution-ReLU layer with *k* filters with Batch Normalization. *GS* is a gating signal which is simply a 1 x 1 Convolution-ReLU layer with *k* filters with Batch Normalization. Finally, *AGk* denotes the soft attention gates with *k* filters that identifies relevant spatial information for each pixel from the low-level feature maps (Lin & Hui, 2018; Oktay et al., 2018) and propagate it to the decoding phase of the network.

The encoder phase of the network consists of:

AP, {c3s1tmax – 32}, {c3s1B – 64t}, {c3s1tmax – 64}, {c3s1B – 128t}, {c3s1tmax – 128}, {c3s1B - 256t}, {c3s1tmax – 256}

The bridge between the encoder and decoder is: {*c7s1tmax – 512*}

The decoder phase of the network consists of:

GS, AG128tUP32s2, GS, AG64tUP64s2, GS, AG32tUP32s2, tUP32s2, {c3s1tmax – 256}, {c3s1tmax – 128}, {c3s1tmax – 64}, {c3s1tmax – 32}, {c1s1B – 1}, {c1s1B – 1}, {c1s1B – 1}, {c1s1B – 1}

More information regarding the Frequency Area Distribution curves

Apart from that, FAD has also been used to (1) quantify total denudation caused by the landslides (Hovius, Stark, & Allen, 1997), (2) estimate landslide hazard (Guzzetti, Reichenbach, Cardinali, Galli, & Ardizzone, 2005), and (3) assess the quality of the mapped inventory (Tanyaş, van Westen, Allstadt, & Jibson, 2019). The Double Pareto function (Equation 8) and the Inverse Gamma function have both been used to quantitatively characterize the probability density function (pdf) distributions of landslides along the range of sizes above and below the rollover (Stark & Hovius, 2001; Malamud et al., 2004). The frequency density versus size distribution, which is the result of the probability density and the total number of occurrences, is examined in certain studies (e.g., (Hergarten, 2002)).

References

Guzzetti, F., Reichenbach, P., Cardinali, M., Galli, M., & Ardizzone, F. (2005). Probabilistic landslide hazard assessment at the basin scale. *Geomorphology*, *72*(1–4), 272–299. https://doi.org/10.1016/j.geomorph.2005.06.002

Hergarten, S. (2002). SOC in Landslides. In *Self-Organized Criticality in Earth Systems*.

Hovius, N., Stark, C. P., & Allen, P. A. (1997). Sediment flux from a mountain belt derived by landslide mapping. *Geology*, *25*(3), 231–234. https://doi.org/10.1130/0091-7613(1997)025<0231:SFFAMB>2.3.CO;2

Johnson, J., Alahi, A., & Fei-Fei, L. (2016). Perceptual losses for real-time style transfer and super-resolution. *Lecture Notes in Computer Science (Including Subseries Lecture Notes in Artificial Intelligence and Lecture Notes in Bioinformatics)*, *9906 LNCS*, 694–711. Springer Verlag. https://doi.org/10.1007/978-3-319-46475-6_43

Lin, S., & Hui, P. (2018). *Where's YOUR focus: Personalized Attention*. https://doi.org/10.48550/arxiv.1802.07931

Malamud, B. D., Turcotte, D. L., Guzzetti, F., & Reichenbach, P. (2004). Landslide inventories and their statistical properties. *Earth Surface Processes and Landforms*, *29*(6), 687–711. https://doi.org/10.1002/esp.1064

Oktay, O., Schlemper, J., Folgoc, L. Le, Lee, M., Heinrich, M., Misawa, K., … Rueckert, D. (2018). *Attention U-Net: Learning Where to Look for the Pancreas*. https://doi.org/10.48550/arxiv.1804.03999

Stark, C. P., & Hovius, N. (2001). The characterization of landslide size distributions. *Geophysical Research Letters*, *28*(6), 1091–1094. https://doi.org/10.1029/2000GL008527

Tanyaş, H., van Westen, C. J., Allstadt, K. E., & Jibson, R. W. (2019). Factors controlling landslide frequency–area distributions. *Earth Surface Processes and Landforms*, *44*(4), 900–917. https://doi.org/10.1002/esp.4543
